# Admission Serum Ionized and Total Calcium as New Predictors of Mortality in Patients with Cardiogenic Shock

**DOI:** 10.1155/2021/6612276

**Published:** 2021-04-08

**Authors:** Yue Yu, Jingwen Yu, Renqi Yao, Pei Wang, Yufeng Zhang, Jian Xiao, Zhinong Wang

**Affiliations:** ^1^Department of Cardiothoracic Surgery, Changzheng Hospital, Naval Medical University, Shanghai 200003, China; ^2^Translational Medicine Research Center, Fourth Medical Center and Medical Innovation Research Division of the Chinese PLA General Hospital, Beijing 100048, China; ^3^Department of Burn Surgery, Changhai Hospital, Naval Medical University, Shanghai 200433, China

## Abstract

**Background:**

Although serum calcium has been proven to be a predictor of mortality in a wide range of diseases, its prognostic value in critically ill patients with cardiogenic shock (CS) remains unknown. This retrospective observational study is aimed at investigating the association of admission calcium with mortality among CS patients.

**Methods:**

Critically ill patients diagnosed with CS in the Medical Information Mart for Intensive Care-III (MIMIC-III) database were included in our study. The study endpoints included 30-day, 90-day, and 365-day all-cause mortalities. First, admission serum ionized calcium (iCa) and total calcium (tCa) levels were analyzed as continuous variables using restricted cubic spline Cox regression models to evaluate the possible nonlinear relationship between serum calcium and mortality. Second, patients with CS were assigned to four groups according to the quartiles (Q1-Q4) of serum iCa and tCa levels, respectively. In addition, multivariable Cox regression analyses were used to assess the independent association of the quartiles of iCa and tCa with clinical outcomes.

**Results:**

A total of 921 patients hospitalized with CS were enrolled in this study. A nonlinear relationship between serum calcium levels and 30-day mortality was observed (all *P* values for nonlinear trend < 0.001). Furthermore, multivariable Cox analysis showed that compared with the reference quartile (Q3: 1.11 ≤ iCa < 1.17 mmol/L), the lowest serum iCa level quartile (Q1: iCa < 1.04 mmol/L) was independently associated with an increased risk of 30-day mortality (Q1 vs. Q3: HR 1.35, 95% CI 1.00-1.83, *P* = 0.049), 90-day mortality (Q1 vs. Q3: HR 1.36, 95% CI 1.03-1.80, *P* = 0.030), and 365-day mortality (Q1 vs. Q3: HR 1.28, 95% CI 1.01-1.67, *P* = 0.046) in patients with CS.

**Conclusions:**

Lower serum iCa levels on admission were potential predictors of an increased risk of mortality in critically ill patients with CS.

## 1. Introduction

Cardiogenic shock (CS) is a severely diminished-cardiac-output state resulting in life-threatening end-organ hypoperfusion and hypoxia [1, 2]. There are numerous causes of CS, including acute myocardial infarction (AMI), severe myocarditis, and end-stage dilated cardiomyopathy [3]. In addition, CS is the most common cause of death for patients hospitalized with AMI [4]. Despite advances in treatment, the in-hospital mortality remains unacceptably high (27%-51%) [5–7]. As mortality peaks within the first 48 hours after CS onset, it is necessary to find an accurate yet user-friendly predictor for early risk stratification to provide more accurate prognostic information and help implement appropriate treatment [8].

Serum calcium plays an essential role in a range of biological processes related to cardiovascular diseases, including myocardial contraction and relaxation, nerve transmission, vascular smooth muscle contractile activity, platelet adhesion, and blood coagulation [9–11]. Thus, alterations in serum calcium concentrations might interfere with myocardial function and cause severe cardiovascular complications and organ dysfunctions [12]. Derangement in serum calcium is known to be extremely common in the intensive care unit (ICU) setting, and several previous studies have shown that increased or/and decreased levels of serum calcium were independent risk predictors for mortality in patients with AMI [13–18], heart failure [19], acute kidney injury (AKI) [20], and acute stroke [21] or individuals in the general population [22–24]; they were also tightly related to cardiovascular risk factors such as hyperlipidemia, hyperglycemia, and hypertension [10, 16].

To the best of our knowledge, there have been no epidemiological studies exploring the prognostic value of serum calcium among critically ill patients with CS. As a common urgent critical illness, patients with CS are at greater risks of kidney injury, impaired gastrointestinal function, or heightened neurohormonal activation, which could affect serum calcium homeostasis [25–28]; it remains unclear whether abnormalities in calcium levels could affect the prognosis of CS. Additionally, most previous studies only focused on the serum tCa [13–15, 21–23, 29–31]. Considering the limitations of tCa measurements in the identification of true calcium derangements (i.e., its dependency on serum albumin levels) [31–33], the prognostic ability of serum iCa was also explored in this study.

In the present study, we aimed to investigate the possible association of admission serum iCa and tCa levels with the risks of all-cause mortality in patients with CS.

## 2. Methods

### 2.1. Study Design

This is a single-center retrospective cohort study, and all the relevant data were collected from the Medical Information Mart for Intensive Care-III (MIMIC-III) database. MIMIC-III is a freely accessible and conveniently sized critical care database covering over 50,000 hospital admissions comprised of 38,645 adults as well as 7,875 neonates admitted to surgical, trauma surgery, coronary, and cardiac surgery recovery ICUs of Beth Israel Deaconess Medical Center (BIDMC) in Boston from 2001 to 2012 [34, 35]. The MIMIC-III database documents contained high-resolution information from hospital monitoring systems (including laboratory data, medication, and hospital administrative data) and bedside monitoring systems (vital signs, caregivers notes, and radiology reports). We passed the “Protecting Human Research Participants” exam and obtained permission to access the dataset (authorization code: 33281932). Furthermore, we conducted this study in accordance with the STrengthening the Reporting of OBservational studies in Epidemiology (STROBE) statement [36].

### 2.2. Ethical Approval

The establishment of the MIMIC-III database was approved by the Institutional Review Boards (IRB) of the Massachusetts Institute of Technology (No. 0403000206) and BIDMC (2001-P-001699/14). Our study utilized the anonymous data available from this database, and hence, the requirement for informed consent was waived. In summary, the study complied with the ethical standards laid down in the 1964 Declaration of Helsinki and its later amendments.

### 2.3. Study Population

We included all ICU patients (aged ≥ 18 years) with the primary diagnosis of CS using International Classification of Diseases, ninth version- (ICD-) 9 diagnosis codes (ICD-9 codes: 785.51) in the MIMIC-III database. Only the data of each patient's first ICU admission were used in this study. Patients were excluded if they had (1) a secondary diagnosis of hepatic dysfunction, renal failure, acute or chronic pancreatitis, parathyroid diseases, or malignancy on admission; (2) a length of stay in the ICU less than 24 hours; (3) incomplete or unobtainable data of serum iCa and tCa measured during the first 24 hours admission; (4) incomplete follow-up information; or (5) more than 10% of individual data missing.

### 2.4. Data Extraction, Preparation, and Definitions

Demographics, vital signs, laboratory tests, medications, and others were extracted from the MIMIC-III database using structured query language (SQL) with PostgreSQL (version 9.4.6, http://www.postgresql.org). The code that supports the MIMIC-III documentation and website is publicly available, and contributions from the community of users are encouraged (https://github.com/MIT-LCP/mimic-website).

Baseline demographic variables included age, sex, ethnicity (white or others), and current smoking status (by Natural Language Processing searches in provider notes, categorized as “yes,” or “no/unknown”). We extracted data on the following comorbidities: coronary artery disease (CAD), chronic heart failure (CHF), atrial fibrillation (AF), hypertension, peripheral artery disease (PAD), stroke, diabetes mellitus (DM), and chronic kidney disease (CKD). Vital signs on admission included systolic blood pressure (SBP), diastolic blood pressure (DBP), mean blood pressure (MBP), and heart rate (HR). Laboratory-based data included iCa, tCa, phosphorus, potassium, sodium, chloride, bicarbonate, lactate, anion gap (AG), pH, creatinine, estimated glomerular filtration rate (eGFR), hemoglobin, platelet, and white blood cell count (WBC). The eGFR was calculated using the Chronic Kidney Disease Epidemiology Collaboration (CKD-Epi) formula [37]. If patients received a laboratory test more than once during their hospitalization, only the initial test results were included for analysis. Three scoring systems (the Sequential Organ Failure Assessment (SOFA), the Simplified Acute Physiology Score II (SAPS II), and the Glasgow Coma Scale (GCS)) were calculated within the first 24 hours after admission using the values associated with the greatest severity of illness. In addition, treatment information data were also collected, including mechanical ventilation, renal replacement treatment (RRT), and in-hospital medication (inotrope and vasoconstrictor) administration.

### 2.5. Identification of Cut-Off Values for Serum iCa and tCa Levels

Serum calcium levels were categorized into four groups according to the quartiles (Q1-Q4) of their concentrations.

### 2.6. Study Outcomes

The primary outcome of our study was 30-day all-cause mortality. Secondary outcomes included 90-day and 365-day all-cause mortality. Patients with missing survival outcome information were excluded from the final cohort.

### 2.7. Statistical Analysis

The data distribution was examined using the Kolmogorov-Smirnov test. Continuous variables are presented as mean (standardized differences (SD)) or median (interquartile range (IQR)) and categorical variables as total number and percentage. Baseline characteristics of enrolled participants were presented by using either Student *t*-test, Kruskal Wallis rank test, Pearson's *χ*^2^ test, or Fisher's exact test as appropriate.

Restricted cubic spline Cox regression models were used to evaluate the possible nonlinear relationship between serum calcium levels and 30-day all-cause mortality [38]. If the test for nonlinearity was not significant, the test result for overall association and linearity was checked, with significant results indicating linear associations.

The Kaplan-Meier method was used to plot unadjusted survival curves, and the log-rank test was used to compare differences between the quartiles of serum calcium. Moreover, Cox proportional hazards regression analysis was performed to examine the relationship between baseline covariates and each endpoint. We separately included the serum iCa and tCa quartiles in multivariable Cox regression models, adjusting for the potential confounders selected based on *P* ≤ 0.05 in the univariable analysis. The third quartile (Q3: 1.11 ≤ iCa < 1.17 mmol/L; 8.3 ≤ tCa < 8.9 mg/dL) was used as a reference group, and the results are presented as hazard ratios (HRs) with 95% confidence intervals (CIs). Furthermore, subgroup analyses were performed to investigate the association between serum calcium levels and mortality. Moreover, most commonly, CS is an emergency disease characterized by unacceptably high in-hospital mortality; therefore, we mainly focused on the short-term mortality of CS and performed subgroup analyses only for the 30-day mortality.

As extensive missing data might lead to bias, variables with over 20% missing values were not included in the subsequent analyses. Correspondingly, multivariate imputation (MI) was used for variables with less than 20% missing values [39, 40]. Variables for which MI was adopted included SBP, DBP, MBP, HR, lactate, AG, pH, and GCS.

A two-tailed *P* value of less than 0.050 was considered to be statistically significant. All statistical analyses were performed using SPSS software (version 22.0; IBM Corporation, St. Louis, Missouri, USA) and R software (version.3.6.1; The R Project for Statistical Computing, TX, USA; http://www.r-project.org).

## 3. Results

### 3.1. Subject and Variable Characteristics

**Figure 1:**
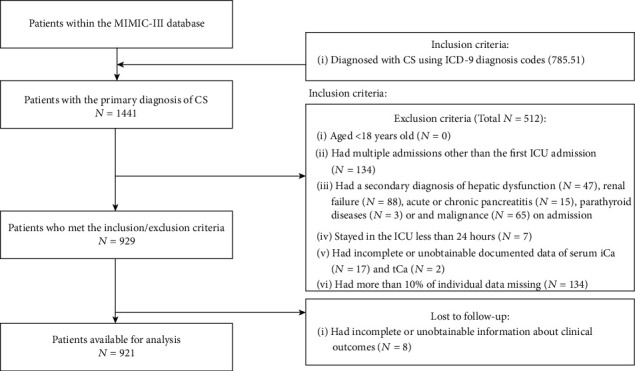
Flow chart of cohort selection. Abbreviation: MIMIC-III: Medical Information Mart for Intensive Care-III; CS: cardiogenic shock; ICD: International Classification of Diseases; ICU: intensive care unit.

After application of the inclusion and exclusion criteria, the final study cohort consisted of 921 CS patients (Figure 1). The median age of the study cohort was 72 (62-81) years, and 60.3% (555/921) subjects were male. The median admission serum iCa and tCa were 1.11 (1.04-1.17) mmol/L and 8.3 (7.8-8.9) mg/dL, respectively.

In the current study, serum tCa levels were divided into the Q1 group (tCa < 7.8 mg/dL), Q2 group (7.8 ≤ tCa < 8.3 mg/dL), Q3 group (8.3 ≤ tCa < 8.9 mg/dL), and Q4 group (8.9 mg/dL ≤ tCa). Similarly, serum iCa levels were divided into the Q1 group (iCa < 1.04 mmol/L), Q2 group (1.04 ≤ iCa < 1.11 mmol/L), Q3 group (1.11 ≤ iCa < 1.17 mmol/L), and Q4 group (1.17 mmol/L ≤ iCa). A total of 223 patients were in the Q1 group (iCa < 1.04 mmol/L), 224 patients were in the Q2 group (1.04 ≤ iCa < 1.11 mmol/L), 231 patients were in the Q3 group (1.11 ≤ iCa < 1.17 mmol/L), and 243 patients were in the Q4 group (1.17 mmol/L ≤ iCa). The comparison of baseline characteristics stratified by serum iCa quartiles is summarized in Table 1. Compared to those in the Q2-4 groups, patients in the Q1 group (iCa < 1.04 mmol/L) had lower SBP (*P* = 0.021), lower tCa concentration (*P* < 0.001), lower bicarbonate concentration (*P* = 0.003), and higher lactate concentration (*P* < 0.001) (Table 1); they also were more likely to receive RRT (*P* < 0.001) (Table 1). Characteristics including age, sex, comorbidities, and scoring systems were relatively flat across each group (Table 1).

### 3.2. Relationship between Serum Calcium Levels and Mortality

**Figure 2:**
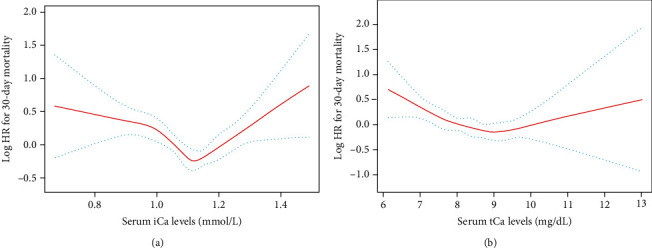
Association of admission serum calcium levels with 30-day mortality in restricted cubic spline models. (a) Serum iCa levels and mortality (b) Serum tCa levels and mortality. The red and blue lines represent the estimated Log HR and the 95% CI, respectively. Abbreviation: HR: hazard ratio; CI: confidence interval; iCa: ionized calcium; tCa: total calcium.

Restricted cubic spline analyses showed the nonlinear relationships between serum calcium levels (iCa and tCa) and the risk of 30-day mortality. (all *P* values for nonlinear trend < 0.001; Figure 2). In addition, we also observed that the lowest risk of mortality was associated with approximately 1.10 mmol/L for iCa and 9.0 mg/dL for tCa.

### 3.3. Survival Analysis

Among the 921 CS patients included, 39.1% (360/921) died during the first 30 days, 47.9% (441/921) died during the first 90 days, and 56.0% (516/921) died during the one-year follow-up period. The 30-day mortality was 48.8% in serum iCa of <1.04 mmol/L, 35.3% in 1.04-1.11 mmol/L, 33.3% in 1.11-1.17 mmol/L, and 39.9% in ≥1.17 mmol/L.

**Figure 3:**
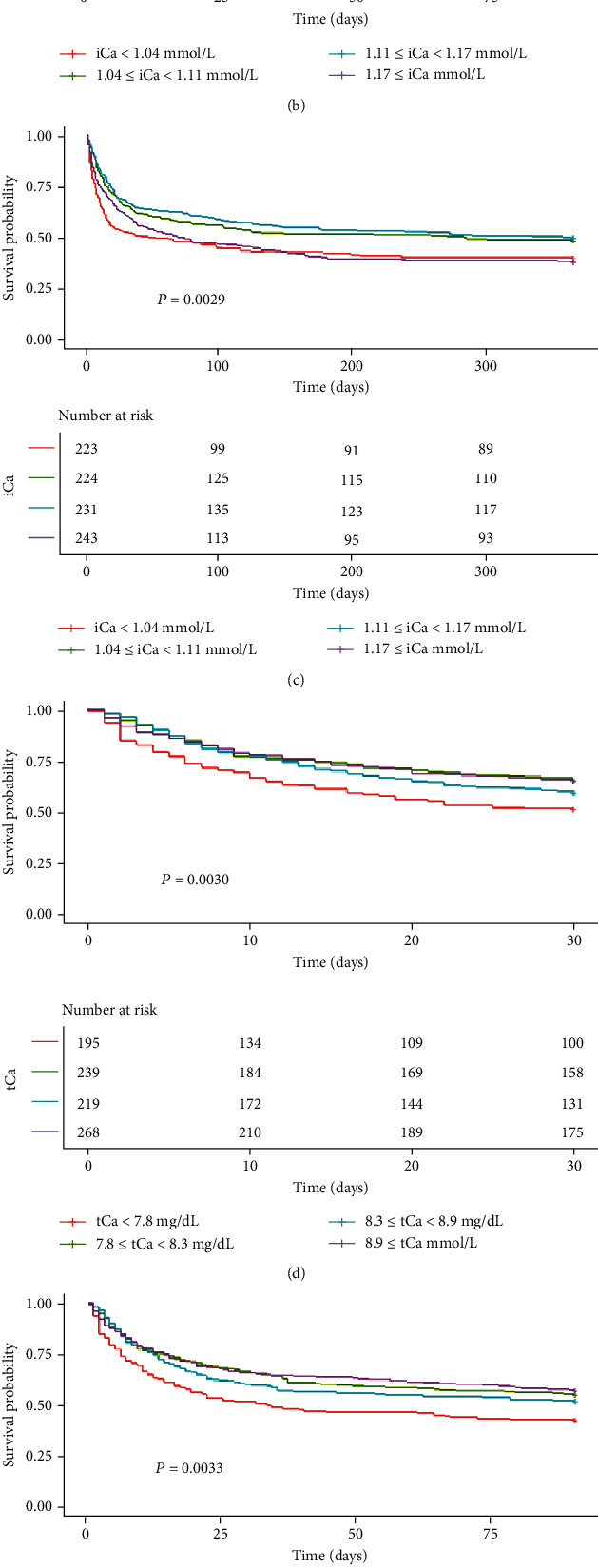
Kaplan–Meier curves of serum calcium level quartiles. (a) Serum iCa and 30-day mortality, (b) serum iCa and 90-day mortality, (c) serum iCa and 365-day mortality, (d) serum tCa and 30-day mortality, (e) serum tCa and 90-day mortality, and (f) serum tCa and 365-day mortality. iCa: ionized calcium; tCa: total calcium. (a–c) The red line represents iCa < 1.04 mmol/L; the green line represents 1.04 ≤ iCa < 1.11 mmol/L; the blue line represents 1.11 ≤ iCa < 1.17 mmol/L; the purple line represents 1.11 ≤ iCa < 1.17 mmol/L. (d–f) The red line represents tCa < 7.8 mg/dL; the green line represents 7.8 ≤ tCa < 8.3 mg/dL; the blue line represents 8.3 ≤ tCa < 8.9 mg/dL; the purple line represents 8.9 ≤ tCa mg/dL.

Kaplan–Meier curves for all-cause death according to the quartiles of serum calcium are shown in Figure 3. The curves of the quartiles of calcium differed significantly (log-rank test: *P* < 0.050 for 30-day, 90-day, and 365-day all-cause mortalities), and patients in the lowest serum calcium quartile had the highest cumulative incidence of mortality.

In the Cox regression analysis, we analyzed serum iCa and tCa concentrations stratified by quartiles to determine whether serum calcium was associated with all-cause mortality (Table 2). The univariable Cox regression models showed that the lowest serum iCa level quartile (iCa < 1.04 mmol/L) and the lowest serum tCa level quartile (tCa < 7.8 mg/dL) were significant predictors of 30-day, 90-day, and 365-day mortalities compared with the reference group (iCa: 1.06-1.14 mmol/L; tCa: 7.9-8.7 mg/dL) (Table 2; Table S1-6). Furthermore, after adjusting for more confounding factors including age, SBP, DBP, MBP, phosphorus, potassium, chloride, bicarbonate, lactate, AG, creatinine, eGFR, WBC, SOFA, SAPS II, and vasopressor use, only the lowest serum iCa level (iCa < 1.04 mmol/L) remained an independent predictor of 30-day mortality (HR 1.35, 95% CI 1.00-1.83, *P* = 0.049), 90-day mortality (HR 1.36, 95% CI 1.03-1.80, *P* = 0.030), and 365-day mortality (HR 1.28, 95% CI 1.01-1.67, *P* = 0.046) (Table 2; Table S1-6). Furthermore, the highest serum iCa level quartile (1.17 mmol/L ≤ iCa) was only associated with 90-day mortality in both the univariable and multivariable Cox regression analyses (Table 2; Table S1-6).

### 3.4. Sensitivity and Subgroup Analysis

We performed subgroup analyses to assess the association between the serum iCa and tCa concentrations and 30-day all-cause mortality (Table 3). Subgroup analyses showed the lowest serum iCa quartile (iCa < 1.04 mmol/L) was also associated with deteriorative mortality in most strata except in patients with a medical history of CHF (*P* = 0.128). In addition, the results of subgroup analyses of serum tCa were shown in Table S7. Moreover, we used original data for analysis without using the MI method, and 807 patients remained in the final cohort. After adjustment for more confounding factors including age, SBP, DBP, MBP, phosphorus, potassium, chloride, bicarbonate, lactate, creatinine, eGFR, SOFA, and SAPS II, the lowest serum iCa level (iCa < 1.04 mmol/L) still remained an independent predictor of 30-day mortality (HR 1.36, 95% CI 1.01-1.85, *P* = 0.047) (Table S8 and S9).

## 4. Discussion

In the present study, we evaluated 921 patients to measure the association of admission serum iCa and tCa levels with all-cause mortality in critically ill patients with CS. Our main findings can be summarized as follows. First, a nonlinear relationship between admission serum calcium (iCa and tCa) and 30-day all-cause mortality could be observed. Second, lower iCa levels (iCa < 1.04 mmol/L) and tCa levels (tCa < 7.8 mg/dL) were associated with an increased risk of 30-day, 90-day, and 365-day mortalities. Third, after adjustments for potential confounding factors, the quartile of the lowest iCa level (iCa < 1.04 mmol/L) remained an independent predictor and was associated with an increase in all-cause mortality. To our knowledge, this study is the first to investigate the prognostic value of serum iCa and tCa levels among critically ill patients with CS.

A considerable number of clinical studies have suggested that the reduced serum calcium level was a common electrolyte disturbance among critically ill patients, which was also associated with increased mortality [41]. Our findings were consistent with the results of studies that evaluated the prognostic value of low serum calcium level in other clinical settings including CAD [14, 15, 18, 42], heart failure [43], AKI [20], CKD [44], trauma [45, 46], coronavirus disease 2019 (COVID-19) [47], or unselected emergency department admissions [48]. Lu et al. [15] reported that lower calcium levels were independent predictors for in-hospital mortality in patients with ST-elevation myocardial infarction (STEMI). Similarly, Yan et al. [14] showed that the baseline serum calcium added an incremental predictive value when combined with the Global Registry of Acute Coronary Events (GRACE) score in acute coronary symptom (ACS) patients. This study was the first to demonstrate that the low serum calcium was also associated with mortality in CS patients. In addition, although the most common cardiac cause of CS is ACS, CS can also result from nonischemic cardiac conditions, and few studies have attempted to explore predictors, which could be applicable to non-ACS presentations [8, 49]. In the subgroup analysis, we found that a lower level of iCa concentration (iCa < 1.04 mmol/L) was a significant predictor of poor prognosis in CS caused by nonischemic cardiac conditions. Consequently, we hope the results of this study will supplement the findings of previous studies. Furthermore, decreased serum calcium levels might imply impaired kidney function [50]. In the present study, the adjustment for eGFR, or stratifying for CS patients according to the medical history of CKD, did not change the significant relationship between decreased serum calcium levels and increased risks of mortality. Thus, our findings showed that a lower serum calcium level might be an independent risk factor for the prognosis of CS rather than a surrogate marker of lower eGFR.

Although the exact mechanisms through which serum calcium leads to an elevated mortality rate remain unclear, there might be several possible explanations for this association. First, severe extracellular hypocalcemia could impact cardiac contractility because the sarcoplasmic reticulum is unable to maintain a sufficient amount of calcium content to initiate myocardial contraction [51]. Second, it has been assumed that the low calcium level might indicate an increased calcium consumption, partially reflecting more plaques or thrombi formed and worsening coronary conditions, resulting in poor outcomes through platelet activation [52]. Third, the appearance of low serum iCa was associated with secondary hyperparathyroidism and increased secretion of parathyroid hormone (PTH), which could promote calcium entry via L-type Ca^2+^ channels with consequent intracellular calcium overloading. Excessive cytosolic Ca^2+^ would affect the cardiac excitation-contraction coupling function, alter autophagic flux, and induce premature activation of intracellular enzymes, all of which contribute to the pathogenesis of CS [53].

Even in the era of reperfusion therapy, CS remains one of the leading causes of death with in-hospital mortality rates still approaching 50% [6, 54]. Individualized and timely risk assessment for each critically ill patient allows a more precise decision-making for therapeutic strategy and medical resource allocation. The prognostic value of several relatively convenient predictors including neutrophil percentage-to-albumin ratio [55], neutrophil-lymphocyte ratio [56], red blood cell distribution width [57], and low diastolic blood pressure [58] was explored. Similarly, even under conditions without imaging or additional laboratory tests, serum calcium could still serve as an effective marker for quick risk assessments. Our findings might provide additional convenience in some special situations, for example, underdeveloped areas. Moreover, further investigations are needed to explore the therapeutic value of serum calcium and find out whether calcium-supplementation therapy in CS patients with low serum calcium could improve their prognosis.

Several limitations of our study should be noted. First, we used data from a single academic medical center in the USA, with the earliest cases from almost 20 years ago, when care may have been inconsistent with currently accepted standards. The single-center nature of the study may also limit the applicability of our findings to other sites. Therefore, multicenter registry and prospective studies are needed to confirm these findings. Second, we measured serum iCa and tCa levels in patients only upon admission to the ICU and did not assess changes during their ICU stay, which might influence the summary results. Third, accurate calcium state determination depends on blood pH levels, because the binding of calcium to protein is particularly pH-sensitive. As pH decreases, H^+^ displaces Ca^2+^ from binding sites, and the amount of iCa increases. Conversely, as the blood pH increases, albumin and the globulins become more negatively charged and bind more calcium, causing the amount of iCa to decrease. Therefore, some sample collection practices (such as prolonged use of a tourniquet or the practice of having the patient clench or pump their fist) can artificially change the pH and cause an inaccurate iCa result, which might influence the results of our study. In addition, although every effort had been made to adjust for confounding factors using multivariate analysis, there remained other unknown factors that confused the prognostic value of serum iCa and tCa.

## 5. Conclusion

Lower serum iCa concentration was an independent predictor of all-cause mortality in critically ill patients with CS. Further studies, especially large prospective studies, are needed to confirm this relationship and validate its clinical significance.

## Figures and Tables

**Table 1:** Characteristics of the study patients according to serum iCa levels.

Characteristics		iCa levels (mmol/L)				
	Total	Q1 (iCa < 1.04)	Q2 (1.04 ≤ iCa < 1.11)	Q3 (1.11 ≤ iCa < 1.17)	Q4 (1.17 ≤ iCa)	*P* value
Number	921	223	224	231	243	
Age (years)	72.0 (62.0-81.0)	73.0 (64.5-81.0)	71.0 (60.8-80.0)	75.0 (63.0-81.5)	72.0 (61.0-80.5)	0.200
Sex (male), *n* (%)	555 (60.3%)	122 (54.7%)	133 (59.4%)	148 (64.1%)	152 (62.6%)	0.181
Ethnicity (white), *n* (%)	630 (68.4%)	142 (63.7%)	162 (72.3%)	163 (70.6%)	163 (67.1%)	0.204
Current smoking, *n* (%)	472 (51.2%)	105 (47.1%)	113 (50.4%)	122 (52.8%)	132 (54.3%)	0.432
ACS etiology, *n* (%)	711 (77.2%)	168 (75.3%)	172 (76.8%)	185 (80.1%)	186 (76.5%)	0.551
Comorbidities, *n* (%)						
CAD	629 (68.3%)	126 (56.5%)	121 (54.0%)	146 (63.2%)	144 (59.3%)	0.181
CHF	205 (22.3%)	53 (23.8%)	56 (25.0%)	51 (22.1%)	45 (18.5%)	0.357
AF	445 (48.3%)	111 (49.8%)	110 (49.1%)	113 (48.9%)	111 (45.7%)	0.812
Hypertension	315 (34.2%)	79 (35.4%)	78 (34.8%)	77 (33.3%)	81 (33.3%)	0.951
PAD	146 (15.9%)	32 (14.4%)	40 (17.9%)	39 (16.9%)	35 (14.4%)	0.655
Stroke	49 (5.3%)	11 (4.9%)	15 (6.7%)	15 (6.5%)	8 (3.3%)	0.318
DM	333 (36.2%)	74 (33.2%)	83 (37.1%)	83 (35.9%)	93 (38.2%)	0.704
CKD	230 (25.0%)	55 (24.7%)	54 (24.1%)	55 (23.8%)	66 (27.2%)	0.828
Vital signs						
SBP (mmHg)	107.0 (97.0-116.0)	104.0 (95.0-113.0)	107.0 (95.8-114.0)	111.0 (99.5-117.0)	107.0 (95.0-117.1)	0.021
DBP (mmHg)	54.0 (47.0-61.0)	54.0 (47.5-61.0)	55.0 (47.0-60.0)	55.0 (47.0-63.0)	54.0 (45.5-61.0)	0.722
MBP (mmHg)	72.0 (64.0-79.0)	71.0 (63.8-78.0)	72.0 (65.6-78.0)	73.0 (65.9-81.0)	72.0 (62.0-79.0)	0.182
HR (beats/min)	87.0 (77.0-99.0)	88.0 (78.0-99.0)	89.5 (78.0-102.0)	86.0 (76.0-99.0)	85.0 (75.0-97.0)	0.058
Laboratory-based data						
tCa (mg/dL)	8.3 (7.8-8.9)	8.0 (7.5-8.7)	8.3 (7.8-8.9)	8.3 (8.0-8.7)	8.7 (8.1-9.2)	<0.001
Phosphorus (mmol/L)	3.8 (3.0-4.9)	4.0 (3.2-5.1)	3.70 (2.9-4.8)	3.7 (3.0-4.7)	4.0 (3.0-5.0)	0.202
Potassium (mmol/L)	3.7 (3.4-4.1)	3.7 (3.4-4.1)	3.7 (3.4-4.0)	3.7 (3.4-4.1)	3.8 (3.4-4.2)	0.438
Sodium (mmol/L)	135.0 (132.0-138.0)	135.0 (132.0-138.0)	135.0 (133.0-138.0)	135.0 (133.0-138.0)	135.0 (132.0-138.0)	0.592
Chloride (mmol/L)	101.0 (97.0-105.0)	102.0 (96.5-105.0)	101.0 (97.0-106.0)	102.0 (97.0-105.0)	102.0 (98.0-106.0)	0.852
Bicarbonate (mmol/L)	20.0 (16.0-23.0)	19.0 (15.0-23.0)	19.0 (16.0-23.0)	21.0 (17.0-24.0)	19.0 (16.0-23.0)	0.003
Lactate (mmol/L)	1.9 (1.3-2.8)	2.1 (1.4-3.4)	1.9 (1.3-2.6)	1.7 (1.1-2.5)	1.8 (1.3-2.6)	<0.001
AG (mmol/L)	14.0 (12.0-17.0)	14.0 (12.0-17.0)	14.0 (12.0-17.0)	14.0 (12.0-16.0)	15.0 (12.0-17.0)	0.050
PH	7.3 (7.2-7.4)	7.3 (7.2-7.4)	7.4 (7.3-7.4)	7.3 (7.2-7.4)	7.3 (7.2-7.4)	0.714
Creatinine (*μ*mol/L)	1.3 (0.0-2.1)	1.3 (0.9-1.9)	1.3 (0.9-2.1)	1.2 (0.9-2.0)	1.3 (1.0-2.5)	0.190
eGFR (mL/min/1.73 m^2^)	60.0 (36.0-87.8)	58.4 (35.1-83.5)	59.9 (35.6-86.8)	63.1 (37.7-94.5)	60.2 (36.9-87.9)	0.234
Hemoglobin (g/dL)	9.7 (8.2-11.3)	9.7 (8.2-11.1)	9.60 (8.0-11.2)	10.0 (8.6-11.6)	9.60 (8.1-11.4)	0.065
Platelet (10^9^/L)	183.0 (127.0-247.0)	171.0 (125.0-230.5)	192.5 (138.8-267.0)	180.0 (128.5-242.5)	189.0 (121.0-254.5)	0.107
WBC (10^9^/L)	10.8 (8.0-14.4)	10.9 (7.5-14.6)	10.9 (8.6-14.3)	10.4 (7.9-13.8)	10.8 (8.1-14.4)	0.553
Scoring system						
SOFA	7.0 (4.0-10.0)	7.0 (5.0-11.0)	7.0 (5.0-10.0)	7.0 (4.0-9.0)	7.0 (5.0-10.0)	0.074
SAPS II	45.0 (35.0-55.0)	47.0 (36.5-57.0)	46.5 (35.8-56.0)	43.0 (32.5-53.5)	45.0 (34.0-54.0)	0.099
GCS	15.0 (14.0-15.0)	15.0 (14.0-15.0)	15.0 (14.0-15.0)	15.0 (14.0-15.0)	15.0 (14.0-15.0)	0.833
Treatment information, *n* (%)						
Mechanical ventilation	738 (80.1%)	183 (82.1%)	181 (80.8%)	180 (77.9%)	194 (79.8%)	0.727
RRT	131 (14.2%)	41 (18.4%)	23 (10.3%)	19 (8.2%)	48 (19.8%)	<0.001
In-hospital medication						
Inotrope use	613 (66.6%)	151 (67.7%)	150 (67.0%)	157 (67.97%)	155 (63.79%)	0.754
Vasopressor use	620 (67.3%)	157 (70.4%)	156 (69.6%)	144 (62.3%)	163 (67.1%)	0.248

CS: cardiogenic shock; iCa: ionized calcium; ACS: acute coronary symptom; CAD: coronary artery disease; CHF: chronic heart failure; PAD: peripheral artery disease; DM: diabetes mellitus; CKD: chronic kidney disease; SBP: systolic blood pressure; DBP: diastolic blood pressure; MBP: mean blood pressure; HR: heart rate; tCa: total calcium; AG: anion gap; eGFR: estimated glomerular filtration rate; WBC: white blood cell; SOFA: Sequential Organ Failure Assessment; SAPS: Simplified Acute Physiology Score; GCS: Glasgow Coma Scale; RRT: renal replacement treatment.

**Table 2:** Association between serum iCa and tCa levels and mortality in patients with CS.

Clinical outcomes	Univariable analysis		Multivariable analysis	
	HR (95% CI)	*P* value	HR (95% CI)	*P* value
30-day mortality				
iCa (mmol/L)				
Q1 (iCa < 1.04)	1.70 (1.27, 2.28)	<0.001	1.35 (1.00, 1.83)	0.049
Q2 (1.04 ≤ iCa < 1.11)	1.07 (0.78, 1.47)	0.657	0.94 (0.68, 1.30)	0.704
Q3 (1.11 ≤ iCa < 1.17)	1		1	
Q4 (1.17 ≤ iCa)	1.29 (0.96, 1.74)	0.096	1.17 (0.86, 1.59)	0.315
tCa (mg/dL)				
Q1 (tCa < 7.8)	1.34 (1.00, 1.79)	0.048	1.29 (0.95, 1.74)	0.097
Q2 (7.8 ≤ tCa < 8.3)	0.84 (0.62, 1.13)	0.250	0.77 (0.56, 1.04)	0.091
Q3 (8.3 ≤ tCa < 8.9)	1		1	
Q4 (8.9 ≤ tCa)	0.84 (0.63, 1.13)	0.252	0.76 (0.56, 1.02)	0.072
90-day mortality				
iCa (mmol/L)				
Q1 (iCa < 1.04)	1.60 (1.22, 2.10)	0.001	1.36 (1.03, 1.80)	0.030
Q2 (1.04 ≤ iCa < 1.11)	1.12 (0.84, 1.49)	0.428	1.04 (0.78, 1.39)	0.785
Q3 (1.11 ≤ iCa < 1.17)	1		1	
Q4 (1.17 ≤ iCa)	1.44 (1.11, 1.88)	0.007	1.33 (1.01, 1.74)	0.041
tCa (mg/dL)				
Q1 (tCa < 7.8)	1.34 (1.03, 1.75)	0.030	1.31 (0.99, 1.72)	0.056
Q2 (7.8 ≤ tCa < 8.3)	0.91 (0.69, 1.19)	0.477	0.83 (0.63, 1.09)	0.179
Q3 (8.3 ≤ tCa < 8.9)	1		1	
Q4 (8.9 ≤ tCa)	0.86 (0.66, 1.12)	0.262	0.79 (0.60, 1.03)	0.086
365-day mortality				
iCa (mmol/L)				
Q1 (iCa < 1.04)	1.45 (1.13, 1.86)	0.003	1.28 (1.01, 1.67)	0.046
Q2 (1.04 ≤ iCa < 1.11)	1.05 (0.81, 1.36)	0.716	0.96 (0.74, 1.26)	0.779
Q3 (1.11 ≤ iCa < 1.17)	1		1	
Q4 (1.17 ≤ iCa)	1.39 (1.09, 1.77)	0.008	1.27 (0.99, 1.63)	0.057
tCa (mg/dL)				
Q1 (tCa < 7.8)	1.28 (1.00, 1.65)	0.050	1.24 (0.95, 1.60)	0.109
Q2 (7.8 ≤ tCa < 8.3)	0.94 (0.73, 1.20)	0.623	0.80 (0.62, 1.03)	0.086
Q3 (8.3 ≤ tCa < 8.9)	1		1	
Q4 (8.9 ≤ tCa)	0.89 (0.70, 1.14)	0.369	0.79 (0.61, 1.02)	0.067

^∗^The confounders from the multivariable Cox regression analyses included age, SBP, DBP, MBP, phosphorus, potassium, chloride, bicarbonate, lactate, AG, creatinine, eGFR, WBC, SOFA, SAPS II, and vasopressor use. CS: cardiogenic shock; iCa: ionized calcium; tCa: total calcium; HR: hazard ratio; CI: confidence interval; SBP: systolic blood pressure; DBP: diastolic blood pressure; MBP: mean blood pressure; AG: anion gap; eGFR: estimated glomerular filtration rate; WBC: white blood cell; SOFA: Sequential Organ Failure Assessment; SAPS: Simplified Acute Physiology Score.

**Table 3:** The association between serum iCa levels and 30-day mortality in the subgroup analysis.

Characteristics		Q1 (iCa < 1.04)	Q2 (1.04 ≤ iCa < 1.11)	Q3 (1.11 ≤ iCa < 1.17)	Q4 (1.17 ≤ iCa)
	*N*	HR (95% CI), *P* value	HR (95% CI), *P* value	Ref.	HR (95% CI), *P* value
Age					
≤72	446	1.87 (1.12, 3.11), 0.016	1.06 (0.61, 1.83), 0.833	1	1.23 (0.73, 2.08), 0.445
>72	475	1.67 (1.16, 2.39), 0.005	1.17 (0.80, 1.72), 0.421	1	1.43 (0.99, 2.06), 0.055
Sex					
Male	555	1.92 (1.29, 2.86), 0.001	1.54 (1.03, 2.29), 0.035	1	1.44 (0.97, 2.13), 0.071
Female	366	1.38 (1.07, 2.14), 0.043	0.60 (0.35, 1.01), 0.054	1	1.10 (0.69, 1.75), 0.691
Current smoking					
No	449	1.57 (1.02, 2.43), 0.041	1.06 (0.67, 1.69), 0.800	1	1.50 (0.97, 2.33), 0.070
Yes	472	1.86 (1.25, 2.77), 0.002	1.10 (0.72, 1.69), 0.656	1	1.13 (0.75, 1.70), 0.555
Etiology					
ACS	620	1.71 (1.19, 2.46), 0.004	1.05 (0.71, 1.55), 0.803	1	1.31 (0.90, 1.89), 0.154
Others	301	1.64 (1.03, 2.71), 0.043	1.10 (0.64, 1.88), 0.726	1	1.24 (0.74, 2.08), 0.413
CAD					
No	629	1.18 (0.69, 2.04), 0.545	0.78 (0.44, 1.39), 0.400	1	1.28 (0.75, 2.18), 0.36
Yes	292	1.99 (1.40, 2.82), <0.001	1.24 (0.85, 1.80), 0.262	1	1.28 (0.89, 1.84), 0.182
CHF					
No	716	1.73 (1.24, 2.41), 0.001	1.09 (0.76, 1.56), 0.6276	1	1.36 (0.97, 1.90), 0.0701
Yes	205	1.61 (0.87, 2.99), 0.128	1.02 (0.53, 1.96), 0.9580	1	0.99 (0.50, 1.99), 0.9858
AF					
No	476	2.02 (1.31, 3.11), 0.001	1.45 (0.93, 2.27), 0.105	1	1.55 (1.01, 2.39), 0.047
Yes	445	1.44 (1.06, 2.15), 0.045	0.79 (0.51, 1.23), 0.299	1	1.08 (0.71, 1.63), 0.731
Hypertension					
No	606	1.44 (1.01, 2.05), 0.042	0.95 (0.65, 1.38), 0.791	1	1.32 (0.93, 1.86), 0.120
Yes	315	2.40 (1.40, 4.12), 0.002	1.40 (0.79, 2.50), 0.253	1	1.22 (0.67, 2.21), 0.510
PAD					
No	775	1.65 (1.19, 2.28), 0.003	1.04 (0.73, 1.47), 0.843	1	1.32 (0.95, 1.83), 0.100
Yes	146	2.02 (1.01, 4.04), 0.046	1.23 (0.61, 2.50), 0.565	1	1.14 (0.55, 2.40), 0.721
DM					
No	588	1.72 (1.19, 2.47), 0.004	1.15 (0.78, 1.70), 0.481	1	1.25 (0.85, 1.83), 0.249
Yes	333	1.67 (1.02, 2.74), 0.041	0.95 (0.56, 1.61), 0.853	1	1.34 (0.83, 2.18), 0.235
CKD					
No	691	1.82 (1.31, 2.54), <0.001	1.06 (0.74, 1.52), 0.753	1	1.31 (0.93, 1.85), 0.128
Yes	230	1.76 (1.03, 2.42), 0.033	1.12 (0.59, 2.11), 0.731	1	1.24 (0.68, 2.27), 0.477
eGFR					
≤60	565	1.58 (1.12, 2.24), 0.009	1.05 (0.73, 1.51), 0.808	1	1.36 (0.96, 1.92), 0.088
>60	356	1.75 (1.01, 3.04), 0.047	0.94 (0.50, 1.75), 0.839	1	1.04 (0.58, 1.86), 0.905

CS: cardiogenic shock; *N*: number; iCa: ionized calcium; HR: hazard ratio; CI: confidence interval; ACS: acute coronary symptom; CAD: coronary artery disease; CHF: chronic heart failure; AF: atrial fibrillation; PAD: peripheral artery disease; DM: diabetes mellitus; CKD: chronic kidney disease; eGFR: estimated glomerular filtration rate.

## Data Availability

The data used to support the findings of this study are available from the corresponding author upon request.

## Abbreviations

CS: Cardiogenic shock
AMI: Acute myocardial infarction
ICU: Intensive care unit
AKI: Acute kidney injury
tCa: Total calcium
iCa: Ionized calcium
MIMIC-III: Medical Information Mart for Intensive Care-III
BIDMC: Beth Israel Deaconess Medical Center
STROBE: STrengthening the Reporting of OBservational studies in Epidemiology
IRB: Institutional Review Boards
ICD: International Classification of Diseases
SQL: Structured query language
CAD: Coronary artery disease
CHF: Chronic heart failure
PAD: Peripheral artery disease
DM: Diabetes mellitus
CKD: Chronic kidney disease
SBP: Systolic blood pressure
DBP: Diastolic blood pressure
MBP: Mean blood pressure
HR: Heart rate
tCa: Total calcium
AG: Anion gap
eGFR: Estimated glomerular filtration rate
WBC: White blood cell
SOFA: Sequential Organ Failure Assessment
SAPS: Simplified Acute Physiology Score
GCS: Glasgow Coma Scale
RRT: Renal replacement treatment
SD: Standardized difference
IQR: Interquartile range
HR: Hazard ratio
CI: Confidence interval
MI: Multivariate imputation
COVID-19: Coronavirus disease 2019
STEMI: ST-elevation myocardial infarction
GRACE: Global Registry of Acute Coronary Events
ACS: Acute coronary symptom
PTH: Parathyroid hormone.

## Supplementary Materials

Supplementary Materials Table S1: univariable and multivariable Cox regression analysis for serum iCa levels and 30-day mortality. Table S2: univariable and multivariable Cox regression analysis for serum iCa levels and 90-day mortality. Table S3: univariable and multivariable Cox regression analysis for serum iCa levels and 365-day mortality. Table S4: univariable and multivariable Cox regression analysis for serum tCa levels and 30-day mortality. Table S5: univariable and multivariable Cox regression analysis for serum tCa levels and 90-day mortality. Table S6: univariable and multivariable Cox regression analysis for serum tCa levels and 365-day mortality. Table S7: the association between serum tCa levels and 30-day mortality in the subgroup analysis. Table S8: univariable and multivariable Cox regression analysis for serum iCa levels and 30-day mortality using original data. Table S9: univariable and multivariable Cox regression analysis for serum tCa levels and 30-day mortality using original data

## References

[1] van Diepen S., Katz J. N., Albert N. M. (2017). Contemporary management of cardiogenic shock: a scientific statement from the American Heart Association. *Circulation*.

[2] Reynolds H. R., Hochman J. S. (2008). Cardiogenic shock: current concepts and improving outcomes. *Circulation*.

[3] Vahdatpour C., Collins D., Goldberg S. (2019). Cardiogenic shock. *Journal of the American Heart Association*.

[4] Thiele H., Zeymer U., Neumann F. J. (2012). Intraaortic balloon support for myocardial infarction with cardiogenic shock. *The New England Journal of Medicine*.

[5] Kolte D., Khera S., Aronow W. S. (2014). Trends in incidence, management, and outcomes of cardiogenic shock complicating ST-elevation myocardial infarction in the United States. *Journal of the American Heart Association*.

[6] Goldberg R. J., Spencer F. A., Gore J. M., Lessard D., Yarzebski J. (2009). Thirty-year trends (1975 to 2005) in the magnitude of, management of, and hospital death rates associated with cardiogenic shock in patients with acute myocardial infarction: a population-based perspective. *Circulation*.

[7] Goldberg R. J., Makam R. C., Yarzebski J., McManus D. D., Lessard D., Gore J. M. (2016). Decade-long trends (2001-2011) in the incidence and hospital death rates associated with the in-hospital development of cardiogenic shock after acute myocardial infarction. *Circulation Cardiovascular Quality and Outcomes*.

[8] Tewelde S. Z., Liu S. S., Winters M. E. (2018). Cardiogenic shock. *Cardiology Clinics*.

[9] Eisner D., Bode E., Venetucci L., Trafford A. (2013). Calcium flux balance in the heart. *Journal of Molecular and Cellular Cardiology*.

[10] Berridge M. J. (2012). Calcium signalling remodelling and disease. *Biochemical Society Transactions*.

[11] Gwathmey J. K., Copelas L., MacKinnon R. (1987). Abnormal intracellular calcium handling in myocardium from patients with end-stage heart failure. *Circulation Research*.

[12] Collage R. D., Howell G. M., Zhang X. (2013). Calcium supplementation during sepsis exacerbates organ failure and mortality via calcium/calmodulin-dependent protein kinase kinase signaling. *Critical Care Medicine*.

[13] Shiyovich A., Plakht Y., Gilutz H. (2018). Serum calcium levels independently predict in-hospital mortality in patients with acute myocardial infarction. *Nutrition, Metabolism, and Cardiovascular Diseases*.

[14] Yan S. D., Liu X. J., Peng Y. (2016). Admission serum calcium levels improve the GRACE risk score prediction of hospital mortality in patients with acute coronary syndrome. *Clinical Cardiology*.

[15] Lu X., Wang Y., Meng H. (2014). Association of admission serum calcium levels and in-hospital mortality in patients with acute ST-elevated myocardial infarction: an eight-year, single-center study in China. *PLoS One*.

[16] Jorde R., Sundsfjord J., Fitzgerald P., Bønaa K. H. (1999). Serum calcium and cardiovascular risk factors and diseases: the Tromsø study. *Hypertension*.

[17] Lind L., Skarfors E., Berglund L., Lithell H., Ljunghall S. (1997). Serum calcium: a new, independent, prospective risk factor for myocardial infarction in middle-aged men followed for 18 years. *Journal of Clinical Epidemiology*.

[18] Gu X., Ding X., Sun H. (2019). Usefulness of serum calcium in the risk stratification of midterm mortality among patients with acute coronary syndrome. *BioMed Research International*.

[19] Jensen A. C., Polcwiartek C., Søgaard P. (2019). The association between serum calcium levels and short-term mortality in patients with chronic heart failure. *The American journal of medicine*.

[20] Wang B., Li D., Gong Y., Ying B., Cheng B. (2019). Association of serum total and ionized calcium with all-cause mortality in critically ill patients with acute kidney injury. *Clinica Chimica Acta*.

[21] Appel S. A., Molshatzki N., Schwammenthal Y. (2011). Serum calcium levels and long-term mortality in patients with acute stroke. *Cerebrovascular Diseases*.

[22] Zhang Z., Xu X., Ni H., Deng H. (2014). Predictive value of ionized calcium in critically ill patients: an analysis of a large clinical database MIMIC II. *PLoS One*.

[23] Wang B., Gong Y., Ying B., Cheng B. (2018). Association of initial serum total calcium concentration with mortality in critical illness. *BioMed Research International*.

[24] Zivin J. R., Gooley T., Zager R. A., Ryan M. J. (2001). Hypocalcemia: a pervasive metabolic abnormality in the critically ill. *American Journal of Kidney Diseases*.

[25] (2018). Renal dysfunction and cardiogenic shock complicating acute coronary syndromes. *European Heart Journal Acute Cardiovascular Care*.

[26] Skowronski G. A., Shaw E. A. P. J., Brooks P. M. (1988). The pathophysiology of shock. *The Medical Journal of Australia*.

[27] Brunkhorst F. M., Clark A. L., Forycki Z. F., Anker S. D. (1999). Pyrexia, procalcitonin, immune activation and survival in cardiogenic shock: the potential importance of bacterial translocation. *International Journal of Cardiology*.

[28] Bushinsky D. A., Monk R. D. (1998). Calcium. *Lancet*.

[29] Egi M., Kim I., Nichol A. (2011). Ionized calcium concentration and outcome in critical illness. *Critical Care Medicine*.

[30] Thongprayoon C., Cheungpasitporn W., Chewcharat A., Mao M. A., Thirunavukkarasu S., Kashani K. B. (2020). Hospital mortality and long-term mortality among hospitalized patients with various admission serum ionized calcium levels. *Postgraduate Medicine*.

[31] Hu Z. D., Huang Y. L., Wang M. Y., Hu G. J. L., Han Y. Q. (2018). Predictive accuracy of serum total calcium for both critically high and critically low ionized calcium in critical illness. *Journal of clinical laboratory analysis*.

[32] Ridefelt P., Helmersson-Karlqvist J. (2017). Albumin adjustment of total calcium does not improve the estimation of calcium status. *Scandinavian Journal of Clinical and Laboratory Investigation*.

[33] D'Orazio P., Visnick H., Balasubramanian S. (2016). Accuracy of commercial blood gas analyzers for monitoring ionized calcium at low concentrations. *Clinica Chimica Acta*.

[34] Goldberger A. L., Amaral L. A., Glass L. (2000). PhysioBank, PhysioToolkit, and PhysioNet: components of a new research resource for complex physiologic signals. *Circulation*.

[35] Johnson A. E., Pollard T. J., Shen L. (2016). MIMIC-III, a freely accessible critical care database. *Scientific Data*.

[36] von Elm E., Altman D. G., Egger M., Pocock S. J., Gøtzsche P. C., Vandenbroucke J. P. (2007). The Strengthening the Reporting of Observational Studies in Epidemiology (STROBE) statement: guidelines for reporting observational studies. *Lancet*.

[37] Levey A. S., Stevens L. A., Schmid C. H. (2009). A new equation to estimate glomerular filtration rate. *Annals of Internal Medicine*.

[38] Desquilbet L., Mariotti F. (2010). Dose-response analyses using restricted cubic spline functions in public health research. *Statistics in Medicine*.

[39] White I. R., Royston P., Wood A. M. (2011). Multiple imputation using chained equations: issues and guidance for practice. *Statistics in Medicine*.

[40] Zhang Z. (2016). Multiple imputation for time series data with Amelia package. *Annals of translational medicine*.

[41] Sauter T. C., Lindner G., Ahmad S. S. (2015). Calcium disorders in the emergency department: independent risk factors for mortality. *PLoS One*.

[42] Chen Q., Zhang Y., Ding D. (2018). Associations between serum calcium, phosphorus and mortality among patients with coronary heart disease. *European Journal of Nutrition*.

[43] Rozentryt P., Niedziela J. T., Hudzik B. (2015). Abnormal serum calcium levels are associated with clinical response to maximization of heart failure therapy. *Polskie Archiwum Medycyny Wewnętrznej*.

[44] Miura S., Yoshihisa A., Takiguchi M. (2015). Association of hypocalcemia with mortality in hospitalized patients with heart failure and chronic kidney disease. *Journal of Cardiac Failure*.

[45] Cherry R. A., Bradburn E., Carney D. E., Shaffer M. L., Gabbay R. A., Cooney R. N. (2006). Do early ionized calcium levels really matter in trauma patients?. *The Journal of Trauma*.

[46] Vinas-Rios J. M., Sanchez-Aguilar M., Sanchez-Rodriguez J. J. (2014). Hypocalcaemia as a prognostic factor of early mortality in moderate and severe traumatic brain injury. *Neurological Research*.

[47] Sun J. K., Zhang W. H., Zou L. (2020). Serum calcium as a biomarker of clinical severity and prognosis in patients with coronavirus disease 2019. *Aging*.

[48] Vroonhof K., van Solinge W. W., Rovers M. M., Huisman A. (2005). Differences in mortality on the basis of laboratory parameters in an unselected population at the emergency department. *Clinical Chemistry and Laboratory Medicine*.

[49] Granger C. B., Goldberg R. J., Dabbous O. (2003). Predictors of hospital mortality in the global registry of acute coronary events. *Archives of Internal Medicine*.

[50] Schwarz S., Trivedi B. K., Kalantar-Zadeh K., Kovesdy C. P. (2006). Association of disorders in mineral metabolism with progression of chronic kidney disease. *Clinical Journal of the American Society of Nephrology*.

[51] Bers D. M. (2002). Cardiac excitation-contraction coupling. *Nature*.

[52] Varga-Szabo D., Braun A., Nieswandt B. (2009). Calcium signaling in platelets. *Journal of Thrombosis and Haemostasis*.

[53] Eisner D. A., Caldwell J. L., Kistamás K., Trafford A. W. (2017). Calcium and excitation-contraction coupling in the heart. *Circulation Research*.

[54] Alexander J. H., Reynolds H. R., Stebbins A. L. (2007). Effect of tilarginine acetate in patients with acute myocardial infarction and cardiogenic Shock. *JAMA*.

[55] Yu Y., Liu Y., Ling X. (2020). The neutrophil percentage-to-albumin ratio as a new predictor of all-cause mortality in patients with cardiogenic shock. *BioMed Research International*.

[56] Peng Y., Wang J., Xiang H. (2020). Prognostic value of neutrophil-lymphocyte ratio in cardiogenic shock: a cohort study. *Medical Science Monitor*.

[57] Wang B., Aihemaiti G., Cheng B., Li X. (2019). Red blood cell distribution width is associated with all-cause mortality in critically ill patients with cardiogenic shock. *Medical Science Monitor*.

[58] Axler O. (2013). Low diastolic blood pressure as best predictor of mortality in cardiogenic shock. *Critical Care Medicine*.

