# Supplementary material for: Admission Serum Ionized and Total Calcium as New Predictors of Mortality in Patients with Cardiogenic Shock
Source: Biomed Res Int. 2021 Apr 8;2021:6612276. doi: 10.1155/2021/6612276 (PMC8049792; doi:10.1155/2021/6612276)
Supplement: Supplementary Materials — Table S1: univariable and multivariable Cox regression analysis for serum iCa levels and 30-day mortality. Table S2: univariable and multivariable Cox regression analysis for serum iCa levels and 90-day mortality. Table S3: univariable and multivariable Cox regression analysis for serum iCa levels and 365-day mortality. Table S4: univariable and multivariable Cox regression analysis for serum tCa levels and 30-day mortality. Table S5: univariable and multivariable Cox regression analysis for serum tCa levels and 90-day mortality. Table S6: univariable and multivariable Cox regression analysis for serum tCa levels and 365-day mortality. Table S7: the association between serum tCa levels and 30-day mortality in the subgroup analysis. Table S8: univariable and multivariable Cox regression analysis for serum iCa levels and 30-day mortality using original data. Table S9: univariable and multivariable Cox regression analysis for serum tCa levels and 30-day mortality using original data [file 6612276.f1.docx]

**Additional Files: Table S1-9**

**Table S1.** Univariable and multivariable Cox regression analysis for serum iCa levels and 30-day mortality

|  | **Univariable analysis** | | **Multivariable analysis** | |
| --- | --- | --- | --- | --- |
| **Characteristics** | **HR (95% CI)** | ***P* value** | **HR (95% CI)** | ***P* value** |
| Age | 1.03 (1.02, 1.04) | <0.001 | 1.03 (1.02, 1.04) | <0.001 |
| Sex |  |  |  |  |
| Male | 1 |  |  |  |
| Female | 1.07 (0.87, 1.32) | 0.539 |  |  |
| Ethnicity |  |  |  |  |
| Others | 1 |  |  |  |
| White | 0.83 (0.67, 1.03) | 0.085 |  |  |
| Current smoking | 1.06 (0.86, 1.31) | 0.574 |  |  |
| ACS etiology | 0.86 (0.69, 1.07) | 0.167 |  |  |
| Comorbidities |  |  |  |  |
| CAD | 1.05 (0.84, 1.32) | 0.654 |  |  |
| CHF | 0.89 (0.69, 1.14) | 0.353 |  |  |
| AF | 1.02 (0.83, 1.25) | 0.873 |  |  |
| Hypertension | 0.85 (0.68, 1.06) | 0.144 |  |  |
| PAD | 1.15 (0.87, 1.50) | 0.322 |  |  |
| Stroke | 0.62 (0.36, 1.09) | 0.095 |  |  |
| DM | 1.01 (0.82, 1.26) | 0.909 |  |  |
| CKD | 0.94 (0.74, 1.19) | 0.608 |  |  |
| Vital signs |  |  |  |  |
| SBP | 0.98 (0.97, 0.99) | <0.001 | 1.00 (0.99, 1.00) | 0.330 |
| DBP | 0.99 (0.98, 0.99) | 0.013 | 1.02 (1.00, 1.03) | 0.026 |
| MBP | 0.99 (0.98, 0.99) | <0.001 | 0.99 (0.98, 1.00) | 0.264 |
| HR | 1.00 (0.99, 1.00) | 0.303 |  |  |
| Laboratory-based data |  |  |  |  |
| iCa |  |  |  |  |
| Q1 (iCa＜1.04) | 1.70 (1.27, 2.28) | <0.001 | 1.35 (1.00, 1.83) | 0.049 |
| Q2 (1.04≤iCa＜1.11) | 1.07 (0.78, 1.47) | 0.657 | 0.94 (0.68, 1.30) | 0.704 |
| Q3 (1.11≤iCa＜1.17) | 1 |  | 1 |  |
| Q4 (1.17≤iCa) | 1.29 (0.96, 1.74) | 0.096 | 1.17 (0.86, 1.59) | 0.315 |
| Phosphorus | 1.19 (1.13, 1.26) | <0.001 | 1.14 (1.07, 1.21) | <0.001 |
| Potassium | 1.30 (1.09, 1.55) | 0.003 | 1.13 (0.93, 1.35) | 0.214 |
| Sodium | 0.99 (0.97, 1.01) | 0.479 |  |  |
| Chloride | 0.98 (0.97, 1.00) | 0.043 | 1.00 (0.98, 1.02) | 0.778 |
| Bicarbonate | 0.94 (0.92, 0.96) | <0.001 | 1.00 (0.98, 1.03) | 0.952 |
| Lactate | 1.19 (1.15, 1.23) | <0.001 | 1.10 (1.04, 1.16) | 0.001 |
| AG | 1.10 (1.08, 1.13) | <0.001 | 1.04 (1.01, 1.08) | 0.013 |
| PH | 0.62 (0.28, 1.37) | 0.234 |  |  |
| Creatinine | 1.10 (1.04, 1.15) | <0.001 | 0.98 (0.88, 1.08) | 0.674 |
| eGFR | 0.99 (0.98, 0.99) | <0.001 | 1.00 (0.99, 1.00) | 0.410 |
| Hemoglobin | 1.02 (0.97, 1.07) | 0.409 |  |  |
| Platelet | 1.00 (1.00, 1.00) | 0.387 |  |  |
| WBC | 1.01 (1.00, 1.02) | 0.045 | 1.01 (1.00, 1.02) | 0.106 |
| Scoring system |  |  |  |  |
| SOFA | 1.10 (1.07, 1.14) | <0.001 | 1.00 (0.95, 1.05) | 0.929 |
| SAPS II | 1.04 (1.03, 1.04) | <0.001 | 1.02 (1.01, 1.03) | 0.001 |
| GCS | 0.98 (0.95, 1.02) | 0.309 |  |  |
| Treatment information |  |  |  |  |
| Mechanical ventilation | 1.13 (0.87, 1.47) | 0.373 |  |  |
| RRT | 1.12 (0.87, 1.44) | 0.371 |  |  |
| In-hospital medication |  |  |  |  |
| Inotrope use | 1.15 (0.92, 1.43) | 0.233 |  |  |
| Vasopressor use | 1.37 (1.09, 1.73) | 0.007 | 1.18 (0.93, 1.51) | 0.167 |

**Abbreviation**: CS: Cardiogenic shock; iCa: Ionized calcium; HR: Hazard ratio; CI: Confidence interval; ACS: Acute coronary symptom; CAD: Coronary artery disease; CHF: Chronic heart failure; PAD: Peripheral artery disease; DM: Diabetes mellitus; CKD: Chronic kidney disease; SBP: Systolic blood pressure; DBP: Diastolic blood pressure; MBP: Mean blood pressure; HR: Heart rate; tCa: Total calcium; AG: Anion gap; eGFR: estimated glomerular filtration rate; WBC: White blood cell; SOFA: Sequential Organ Failure Assessment; SAPS: Simplified Acute Physiology Score; GCS: Glasgow Coma Scale; RRT: Renal replacement treatment.

**Table S2.** Univariable and multivariable Cox regression analysis for serum iCa levels and 90-day mortality

|  | **Univariable analysis** | | **Multivariable analysis** | |
| --- | --- | --- | --- | --- |
| **Characteristics** | **HR (95% CI)** | ***P* value** | **HR (95% CI)** | ***P* value** |
| Age | 1.03 (1.02, 1.04) | <0.001 | 1.03 (1.02, 1.04) | <0.001 |
| Sex, |  |  |  |  |
| Male | 1 |  |  |  |
| Female | 1.05 (0.87, 1.27) | 0.600 |  |  |
| Ethnicity |  |  |  |  |
| Others | 1 |  |  |  |
| White | 0.83 (0.68, 1.00) | 0.056 |  |  |
| Current smoking | 1.13 (0.93, 1.36) | 0.210 |  |  |
| ACS etiology | 0.92 (0.75, 1.12) | 0.386 |  |  |
| Comorbidities |  |  |  |  |
| CAD | 0.99 (0.81, 1.21) | 0.910 |  |  |
| CHF | 0.88 (0.70, 1.11) | 0.289 |  |  |
| AF | 1.04 (0.86, 1.25) | 0.707 |  |  |
| Hypertension | 0.80 (0.65, 0.98) | 0.032 |  |  |
| PAD | 1.05 (0.81, 1.35) | 0.718 |  |  |
| Stroke | 0.60 (0.37, 1.00) | 0.048 |  |  |
| DM | 1.01 (0.83, 1.22) | 0.955 |  |  |
| CKD | 0.92 (0.74, 1.14) | 0.438 |  |  |
| Vital signs |  |  |  |  |
| SBP | 0.98 (0.98, 0.99) | <0.001 | 1.00 (0.99, 1.01) | 0.995 |
| DBP | 0.99 (0.98, 0.99) | 0.004 | 1.00 (0.99, 1.02) | 0.980 |
| MBP | 0.98 (0.98, 0.99) | <0.001 | 0.99 (0.98, 1.00) | 0.053 |
| HR | 1.00 (0.99, 1.01) | 0.878 |  |  |
| Laboratory-based data |  |  |  |  |
| iCa |  |  |  |  |
| Q1 (iCa＜1.04) | 1.60 (1.22, 2.10) | 0.001 | 1.36 (1.03, 1.80) | 0.030 |
| Q2 (1.04≤iCa＜1.11) | 1.12 (0.84, 1.49) | 0.428 | 1.04 (0.78, 1.39) | 0.785 |
| Q3 (1.11≤iCa＜1.17) | 1 |  | 1 |  |
| Q4 (1.17≤iCa) | 1.44 (1.11, 1.88) | 0.007 | 1.33 (1.01, 1.74) | 0.041 |
| Phosphorus | 1.17 (1.11, 1.23) | <0.001 | 1.13 (1.07, 1.20) | <0.001 |
| Potassium | 1.33 (1.13, 1.55) | <0.001 | 1.18 (0.99, 1.39) | 0.059 |
| Sodium | 0.99 (0.97, 1.01) | 0.291 |  |  |
| Chloride | 0.99 (0.97, 1.00) | 0.062 |  |  |
| Bicarbonate | 0.94 (0.93, 0.96) | <0.001 | 1.00 (0.98, 1.02) | 0.880 |
| Lactate | 1.17 (1.12, 1.21) | <0.001 | 1.09 (1.03, 1.14) | 0.002 |
| AG | 1.09 (1.07, 1.11) | <0.001 | 1.04 (1.01, 1.07) | 0.010 |
| PH | 0.68 (0.33, 1.40) | 0.293 |  |  |
| Creatinine | 1.09 (1.04, 1.15) | <0.001 | 0.99 (0.90, 1.08) | 0.830 |
| eGFR | 0.99 (0.99, 0.99) | <0.001 | 1.00 (0.99, 1.00) | 0.888 |
| Hemoglobin | 1.01 (0.97, 1.05) | 0.796 |  |  |
| Platelet | 1.00 (1.00, 1.00) | 0.301 |  |  |
| WBC | 1.01 (1.00, 1.02) | 0.080 |  |  |
| Scoring system |  |  |  |  |
| SOFA | 1.10 (1.07, 1.13) | <0.001 | 1.00 (0.96, 1.04) | 0.990 |
| SAPS II | 1.03 (1.03, 1.04) | <0.001 | 1.02 (1.01, 1.03) | <0.001 |
| GCS | 0.98 (0.95, 1.01) | 0.158 |  |  |
| Treatment information |  |  |  |  |
| Mechanical ventilation | 0.98 (0.78, 1.24) | 0.877 |  |  |
| RRT | 1.07 (0.85, 1.34) | 0.578 |  |  |
| In-hospital medication |  |  |  |  |
| Inotrope use | 0.99 (0.81, 1.21) | 0.939 |  |  |
| Vasopressor use | 1.24 (1.01, 1.52) | 0.037 | 1.09 (0.88, 1.35) | 0.412 |

**Abbreviation**: CS: Cardiogenic shock; iCa: Ionized calcium; HR: Hazard ratio; CI: Confidence interval; ACS: Acute coronary symptom; CAD: Coronary artery disease; CHF: Chronic heart failure; PAD: Peripheral artery disease; DM: Diabetes mellitus; CKD: Chronic kidney disease; SBP: Systolic blood pressure; DBP: Diastolic blood pressure; MBP: Mean blood pressure; HR: Heart rate; tCa: Total calcium; AG: Anion gap; eGFR: estimated glomerular filtration rate; WBC: White blood cell; SOFA: Sequential Organ Failure Assessment; SAPS: Simplified Acute Physiology Score; GCS: Glasgow Coma Scale; RRT: Renal replacement treatment.

**Table S3.** Univariable and multivariable Cox regression analysis for serum iCa levels and 365-day mortality

|  | **Univariable analysis** | | **Multivariable analysis** | |
| --- | --- | --- | --- | --- |
| **Characteristics** | **HR (95% CI)** | ***P* value** | **HR (95% CI)** | ***P* value** |
| Age | 1.03 (1.02, 1.03) | <0.001 | 1.02 (1.02, 1.03) | <0.001 |
| Sex |  |  |  |  |
| Male | 1 |  |  |  |
| Female | 1.11 (0.93, 1.32) | 0.253 |  |  |
| Ethnicity |  |  |  |  |
| Others | 1 |  |  |  |
| White | 0.82 (0.68, 0.98) | 0.030 | 0.80 (0.66, 0.97) | 0.022 |
| Current smoking | 1.10 (0.93, 1.31) | 0.263 |  |  |
| ACS etiology | 0.92 (0.77, 1.11) | 0.399 |  |  |
| Comorbidities |  |  |  |  |
| CAD | 0.98 (0.81, 1.17) | 0.801 |  |  |
| CHF | 0.86 (0.70, 1.06) | 0.165 |  |  |
| AF | 1.04 (0.88, 1.24) | 0.629 |  |  |
| Hypertension | 0.77 (0.63, 0.92) | 0.006 | 0.91 (0.75, 1.12) | 0.378 |
| PAD | 1.08 (0.85, 1.36) | 0.529 |  |  |
| Stroke | 0.66 (0.43, 1.02) | 0.064 |  |  |
| DM |  |  |  |  |
| CKD | 0.98 (0.80, 1.19) | 0.814 |  |  |
| Vital signs |  |  |  |  |
| SBP | 0.98 (0.98, 0.99) | <0.001 | 1.00 (0.99, 1.01) | 0.983 |
| DBP | 0.98 (0.97, 0.99) | <0.001 | 1.01 (1.00, 1.02) | 0.121 |
| MBP | 0.98 (0.98, 0.99) | <0.001 | 0.99 (0.98, 1.00) | 0.036 |
| HR | 1.00 (0.99, 1.01) | 0.984 |  |  |
| Laboratory-based data |  |  |  |  |
| iCa |  |  |  |  |
| Q1 (iCa＜1.04) | 1.45 (1.13, 1.86) | 0.003 | 1.28 (1.01, 1.67) | 0.046 |
| Q2 (1.04≤iCa＜1.11) | 1.05 (0.81, 1.36) | 0.716 | 0.96 (0.74, 1.26) | 0.779 |
| Q3 (1.11≤iCa＜1.17) | 1 |  | 1 |  |
| Q4 (1.17≤iCa) | 1.39 (1.09, 1.77) | 0.008 | 1.27 (0.99, 1.63) | 0.057 |
| Phosphorus | 1.15 (1.09, 1.21) | <0.001 | 1.10 (1.04, 1.16) | 0.001 |
| Potassium | 1.31 (1.13, 1.52) | <0.001 | 1.14 (0.98, 1.34) | 0.090 |
| Sodium | 0.99 (0.98, 1.01) | 0.481 |  |  |
| Chloride | 0.99 (0.97, 1.00) | 0.051 |  |  |
| Bicarbonate | 0.95 (0.93, 0.96) | <0.001 | 1.00 (0.98, 1.02) | 0.959 |
| Lactate | 1.16 (1.12, 1.20) | <0.001 | 1.07 (1.02, 1.12) | 0.006 |
| AG | 1.09 (1.07, 1.11) | <0.001 | 1.04 (1.01, 1.07) | 0.007 |
| PH | 0.68 (0.35, 1.34) | 0.263 |  |  |
| Creatinine | 1.09 (1.05, 1.14) | <0.001 | 0.96 (0.88, 1.05) | 0.347 |
| eGFR | 0.99 (0.99, 0.99) | <0.001 | 1.00 (0.99, 1.00) | 0.251 |
| Hemoglobin | 0.98 (0.94, 1.02) | 0.305 |  |  |
| Platelet | 1.00 (1.00, 1.00) | 0.290 |  |  |
| WBC | 1.01 (1.00, 1.02) | 0.015 | 1.01 (1.00, 1.02) | 0.023 |
| Scoring system |  |  |  |  |
| SOFA | 1.10 (1.07, 1.12) | <0.001 | 1.00 (0.96, 1.04) | 0.971 |
| SAPS II | 1.03 (1.03, 1.04) | <0.001 | 1.02 (1.01, 1.03) | <0.001 |
| GCS | 0.97 (0.95, 1.00) | 0.067 |  |  |
| Treatment information |  |  |  |  |
| Mechanical ventilation |  |  |  |  |
| RRT | 1.08 (0.87, 1.34) | 0.469 |  |  |
| In-hospital medication |  |  |  |  |
| Inotrope use | 1.03 (0.85, 1.23) | 0.779 |  |  |
| Vasopressor use | 1.26 (1.04, 1.52) | 0.017 | 1.12 (0.92, 1.37) | 0.246 |

**Abbreviation**: CS: Cardiogenic shock; iCa: Ionized calcium; HR: Hazard ratio; CI: Confidence interval; ACS: Acute coronary symptom; CAD: Coronary artery disease; CHF: Chronic heart failure; PAD: Peripheral artery disease; DM: Diabetes mellitus; CKD: Chronic kidney disease; SBP: Systolic blood pressure; DBP: Diastolic blood pressure; MBP: Mean blood pressure; HR: Heart rate; tCa: Total calcium; AG: Anion gap; eGFR: estimated glomerular filtration rate; WBC: White blood cell; SOFA: Sequential Organ Failure Assessment; SAPS: Simplified Acute Physiology Score; GCS: Glasgow Coma Scale; RRT: Renal replacement treatment.

**Table S4.** Univariable and multivariable Cox regression analysis for serum tCa levels and 30-day mortality

|  | **Univariable analysis** | | **Multivariable analysis** | |
| --- | --- | --- | --- | --- |
| **Characteristics** | **HR (95% CI)** | ***P* value** | **HR (95% CI)** | ***P* value** |
| Age | 1.03 (1.02, 1.04) | <0.001 | 1.03 (1.02, 1.04) | <0.001 |
| Sex |  |  |  |  |
| Male | 1 |  |  |  |
| Female | 1.07 (0.87, 1.32) | 0.539 |  |  |
| Ethnicity |  |  |  |  |
| Others | 1 |  |  |  |
| White | 0.83 (0.67, 1.03) | 0.085 |  |  |
| Current smoking | 1.06 (0.86, 1.31) | 0.574 |  |  |
| ACS etiology | 0.86 (0.69, 1.07) | 0.167 |  |  |
| Comorbidities |  |  |  |  |
| CAD | 1.05 (0.84, 1.32) | 0.654 |  |  |
| CHF | 0.89 (0.69, 1.14) | 0.353 |  |  |
| AF | 1.02 (0.83, 1.25) | 0.873 |  |  |
| Hypertension | 0.85 (0.68, 1.06) | 0.144 |  |  |
| PAD | 1.15 (0.87, 1.50) | 0.322 |  |  |
| Stroke | 0.62 (0.36, 1.09) | 0.095 |  |  |
| DM | 1.01 (0.82, 1.26) | 0.909 |  |  |
| CKD | 0.94 (0.74, 1.19) | 0.608 |  |  |
| Vital signs |  |  |  |  |
| SBP | 0.98 (0.97, 0.99) | <0.001 | 1.00 (0.99, 1.00) | 0.315 |
| DBP | 0.99 (0.98, 0.99) | 0.013 | 1.02 (1.00, 1.03) | 0.057 |
| MBP | 0.99 (0.98, 0.99) | <0.001 | 0.99 (0.98, 1.00) | 0.185 |
| HR | 1.00 (0.99, 1.00) | 0.303 |  |  |
| Laboratory-based data |  |  |  |  |
| tCa, |  |  |  |  |
| Q1 (tCa＜7.8) | 1.34 (1.00, 1.79) | 0.048 | 1.29 (0.95, 1.74) | 0.097 |
| Q2 (7.8≤tCa＜8.3) | 0.84 (0.62, 1.13) | 0.250 | 0.77 (0.56, 1.04) | 0.091 |
| Q3 (8.3≤tCa＜8.9) | 1 |  | 1 |  |
| Q4 (8.9≤tCa) | 0.84 (0.63, 1.13) | 0.252 | 0.76 (0.56, 1.02) | 0.072 |
| Phosphorus | 1.19 (1.13, 1.26) | <0.001 | 1.15 (1.08, 1.22) | <0.001 |
| Potassium | 1.30 (1.09, 1.55) | 0.003 | 1.07 (0.89, 1.29) | 0.480 |
| Sodium | 0.99 (0.97, 1.01) | 0.479 |  |  |
| Chloride | 0.98 (0.97, 1.00) | 0.043 | 1.00 (0.98, 1.01) | 0.681 |
| Bicarbonate | 0.94 (0.92, 0.96) | <0.001 | 1.00 (0.98, 1.03) | 0.800 |
| Lactate | 1.19 (1.15, 1.23) | <0.001 | 1.11 (1.05, 1.17) | <0.001 |
| AG | 1.10 (1.08, 1.13) | <0.001 | 1.04 (1.01, 1.08) | 0.021 |
| PH | 0.62 (0.28, 1.37) | 0.234 |  |  |
| Creatinine | 1.10 (1.04, 1.15) | <0.001 | 1.00 (0.90, 1.11) | 0.989 |
| eGFR | 0.99 (0.98, 0.99) | <0.001 | 1.00 (0.99, 1.00) | 0.354 |
| Hemoglobin | 1.02 (0.97, 1.07) | 0.409 |  |  |
| Platelet | 1.00 (1.00, 1.00) | 0.387 |  |  |
| WBC | 1.01 (1.00, 1.02) | 0.045 | 1.01 (1.00, 1.02) | 0.153 |
| Scoring system |  |  |  |  |
| SOFA | 1.10 (1.07, 1.14) | <0.001 | 1.00 (0.95, 1.05) | 0.897 |
| SAPS II | 1.04 (1.03, 1.04) | <0.001 | 1.02 (1.01, 1.03) | 0.001 |
| GCS | 0.98 (0.95, 1.02) | 0.309 |  |  |
| Treatment information |  |  |  |  |
| Mechanical ventilation | 1.13 (0.87, 1.47) | 0.373 |  |  |
| RRT | 1.12 (0.87, 1.44) | 0.371 |  |  |
| In-hospital medication |  |  |  |  |
| Inotrope use | 1.15 (0.92, 1.43) | 0.233 |  |  |
| Vasopressor use | 1.37 (1.09, 1.73) | 0.007 | 1.18 (0.93, 1.49) | 0.185 |

**Abbreviation**: CS: Cardiogenic shock; tCa: Total calcium; HR: Hazard ratio; CI: Confidence interval; ACS: Acute coronary symptom; CAD: Coronary artery disease; CHF: Chronic heart failure; PAD: Peripheral artery disease; DM: Diabetes mellitus; CKD: Chronic kidney disease; SBP: Systolic blood pressure; DBP: Diastolic blood pressure; MBP: Mean blood pressure; HR: Heart rate; tCa: Total calcium; AG: Anion gap; eGFR: estimated glomerular filtration rate; WBC: White blood cell; SOFA: Sequential Organ Failure Assessment; SAPS: Simplified Acute Physiology Score; GCS: Glasgow Coma Scale; RRT: Renal replacement treatment.

**Table S5.** Univariable and multivariable Cox regression analysis for serum tCa levels and 90-day mortality

|  | **Univariable analysis** | | **Multivariable analysis** | |
| --- | --- | --- | --- | --- |
| **Characteristics** | **HR (95% CI)** | ***P* value** | **HR (95% CI)** | ***P* value** |
| Age, years | 1.03 (1.02, 1.04) | <0.001 | 1.03 (1.02, 1.04) | <0.001 |
| Sex |  |  |  |  |
| Male | 1 |  |  |  |
| Female | 1.05 (0.87, 1.27) | 0.600 |  |  |
| Ethnicity |  |  |  |  |
| Others | 1 |  |  |  |
| White | 0.83 (0.68, 1.00) | 0.056 |  |  |
| Current smoking | 1.13 (0.93, 1.36) | 0.210 |  |  |
| ACS etiology | 0.92 (0.75, 1.12) | 0.386 |  |  |
| Comorbidities |  |  |  |  |
| CAD | 0.99 (0.81, 1.21) | 0.910 |  |  |
| CHF | 0.88 (0.70, 1.11) | 0.289 |  |  |
| AF | 1.04 (0.86, 1.25) | 0.707 |  |  |
| Hypertension | 0.80 (0.65, 0.98) | 0.032 |  |  |
| PAD | 1.05 (0.81, 1.35) | 0.718 |  |  |
| Stroke | 0.60 (0.37, 1.00) | 0.048 |  |  |
| DM | 1.01 (0.83, 1.22) | 0.955 |  |  |
| CKD | 0.92 (0.74, 1.14) | 0.438 |  |  |
| Vital signs |  |  |  |  |
| SBP | 0.98 (0.98, 0.99) | <0.001 | 1.00 (0.99, 1.01) | 0.970 |
| DBP | 0.99 (0.98, 0.99) | 0.004 | 1.01 (1.00, 1.03) | 0.056 |
| MBP | 0.98 (0.98, 0.99) | <0.001 | 0.99 (0.98, 1.00) | 0.033 |
| HR | 1.00 (0.99, 1.01) | 0.878 |  |  |
| Laboratory-based data |  |  |  |  |
| tCa |  |  |  |  |
| Q1 (tCa＜7.8) | 1.34 (1.03, 1.75) | 0.030 | 1.31 (0.99, 1.72) | 0.056 |
| Q2 (7.8≤tCa＜8.3) | 0.91 (0.69, 1.19) | 0.477 | 0.83 (0.63, 1.09) | 0.179 |
| Q3 (8.3≤tCa＜8.9) | 1 |  | 1 |  |
| Q4 (8.9≤tCa) | 0.86 (0.66, 1.12) | 0.262 | 0.79 (0.60, 1.03) | 0.086 |
| Phosphorus | 1.17 (1.11, 1.23) | <0.001 | 1.14 (1.08, 1.21) | <0.001 |
| Potassium | 1.33 (1.13, 1.55) | <0.001 | 1.13 (0.95, 1.33) | 0.163 |
| Sodium | 0.99 (0.97, 1.01) | 0.291 |  |  |
| Chloride | 0.99 (0.97, 1.00) | 0.062 |  |  |
| Bicarbonate | 0.94 (0.93, 0.96) | <0.001 | 1.00 (0.98, 1.02) | 0.967 |
| Lactate | 1.17 (1.12, 1.21) | <0.001 | 1.09 (1.04, 1.15) | 0.001 |
| AG | 1.09 (1.07, 1.11) | <0.001 | 1.04 (1.01, 1.07) | 0.016 |
| PH | 0.68 (0.33, 1.40) | 0.293 |  |  |
| Creatinine | 1.09 (1.04, 1.15) | <0.001 | 1.02 (0.93, 1.11) | 0.721 |
| eGFR | 0.99 (0.99, 0.99) | <0.001 | 1.00 (0.99, 1.00) | 0.828 |
| Hemoglobin | 1.01 (0.97, 1.05) | 0.796 |  |  |
| Platelet | 1.00 (1.00, 1.00) | 0.301 |  |  |
| WBC | 1.01 (1.00, 1.02) | 0.080 |  |  |
| Scoring system |  |  |  |  |
| SOFA | 1.10 (1.07, 1.13) | <0.001 | 1.00 (0.96, 1.04) | 0.928 |
| SAPS II | 1.03 (1.03, 1.04) | <0.001 | 1.02 (1.01, 1.03) | <0.001 |
| GCS | 0.98 (0.95, 1.01) | 0.158 |  |  |
| Treatment information |  |  |  |  |
| Mechanical ventilation | 0.98 (0.78, 1.24) | 0.877 |  |  |
| RRT | 1.07 (0.85, 1.34) | 0.578 |  |  |
| In-hospital medication |  |  |  |  |
| Inotrope use | 0.99 (0.81, 1.21) | 0.939 |  |  |
| Vasopressor use | 1.24 (1.01, 1.52) | 0.037 | 1.08 (0.88, 1.34) | 0.454 |

**Abbreviation**: CS: Cardiogenic shock; tCa: Total calcium; HR: Hazard ratio; CI: Confidence interval; ACS: Acute coronary symptom; CAD: Coronary artery disease; CHF: Chronic heart failure; PAD: Peripheral artery disease; DM: Diabetes mellitus; CKD: Chronic kidney disease; SBP: Systolic blood pressure; DBP: Diastolic blood pressure; MBP: Mean blood pressure; HR: Heart rate; tCa: Total calcium; AG: Anion gap; eGFR: estimated glomerular filtration rate; WBC: White blood cell; SOFA: Sequential Organ Failure Assessment; SAPS: Simplified Acute Physiology Score; GCS: Glasgow Coma Scale; RRT: Renal replacement treatment.

**Table S6.** Univariable and multivariable Cox regression analysis for serum tCa levels and 365-day mortality

|  | **Univariable analysis** | | **Multivariable analysis** | |
| --- | --- | --- | --- | --- |
| **Characteristics** | **HR (95% CI)** | ***P* value** | **HR (95% CI)** | ***P* value** |
| Age, years | 1.03 (1.02, 1.03) | <0.001 | 1.02 (1.02, 1.03) | <0.001 |
| Sex, |  |  |  |  |
| Male | 1 |  |  |  |
| Female | 1.11 (0.93, 1.32) | 0.253 |  |  |
| Ethnicity |  |  |  |  |
| Others | 1 |  | 1 |  |
| White | 0.82 (0.68, 0.98) | 0.030 | 0.77 (0.64, 0.93) | 0.007 |
| Current smoking | 1.10 (0.93, 1.31) | 0.263 |  |  |
| ACS etiology | 0.92 (0.77, 1.11) | 0.399 |  |  |
| Comorbidities |  |  |  |  |
| CAD | 0.98 (0.81, 1.17) | 0.801 |  |  |
| CHF | 0.86 (0.70, 1.06) | 0.165 |  |  |
| AF | 1.04 (0.88, 1.24) | 0.629 |  |  |
| Hypertension | 0.77 (0.63, 0.92) | 0.006 | 0.87 (0.71, 1.06) | 0.169 |
| PAD | 1.08 (0.85, 1.36) | 0.529 |  |  |
| Stroke | 0.66 (0.43, 1.02) | 0.064 |  |  |
| DM |  |  |  |  |
| CKD | 0.98 (0.80, 1.19) | 0.814 |  |  |
| Vital signs |  |  |  |  |
| SBP | 0.98 (0.98, 0.99) | <0.001 | 1.00 (0.99, 1.01) | 0.970 |
| DBP | 0.98 (0.97, 0.99) | <0.001 | 1.01 (1.00, 1.02) | 0.143 |
| MBP | 0.98 (0.98, 0.99) | <0.001 | 0.99 (0.98, 1.00) | 0.019 |
| HR | 1.00 (0.99, 1.01) | 0.984 |  |  |
| Laboratory-based data |  |  |  |  |
| tCa |  |  |  |  |
| Q1 (tCa＜7.8) | 1.28 (1.00, 1.65) | 0.050 | 1.24 (0.95, 1.60) | 0.109 |
| Q2 (7.8≤tCa＜8.3) | 0.94 (0.73, 1.20) | 0.623 | 0.80 (0.62, 1.03) | 0.086 |
| Q3 (8.3≤tCa＜8.9) | 1 |  | 1 |  |
| Q4 (8.9≤tCa) | 0.89 (0.70, 1.14) | 0.369 | 0.79 (0.61, 1.02) | 0.067 |
| Phosphorus | 1.15 (1.09, 1.21) | <0.001 | 1.11 (1.05, 1.17) | <0.001 |
| Potassium | 1.31 (1.13, 1.52) | <0.001 | 1.11 (0.95, 1.30) | 0.178 |
| Sodium | 0.99 (0.98, 1.01) | 0.481 |  |  |
| Chloride | 0.99 (0.97, 1.00) | 0.051 |  |  |
| Bicarbonate | 0.95 (0.93, 0.96) | <0.001 | 1.00 (0.98, 1.02) | 0.872 |
| Lactate | 1.16 (1.12, 1.20) | <0.001 | 1.07 (1.02, 1.13) | 0.004 |
| AG | 1.09 (1.07, 1.11) | <0.001 | 1.04 (1.01, 1.07) | 0.012 |
| PH | 0.68 (0.35, 1.34) | 0.263 |  |  |
| Creatinine | 1.09 (1.05, 1.14) | <0.001 | 0.98 (0.90, 1.07) | 0.680 |
| eGFR | 0.99 (0.99, 0.99) | <0.001 | 1.00 (0.99, 1.00) | 0.248 |
| Hemoglobin | 0.98 (0.94, 1.02) | 0.305 |  |  |
| Platelet | 1.00 (1.00, 1.00) | 0.290 |  |  |
| WBC | 1.01 (1.00, 1.02) | 0.015 | 1.01 (1.00, 1.02) | 0.029 |
| Scoring system |  |  |  |  |
| SOFA | 1.10 (1.07, 1.12) | <0.001 | 1.00 (0.96, 1.04) | 0.994 |
| SAPS II | 1.03 (1.03, 1.04) | <0.001 | 1.02 (1.01, 1.03) | <0.001 |
| GCS | 0.97 (0.95, 1.00) | 0.067 |  |  |
| Treatment information |  |  |  |  |
| Mechanical ventilation |  |  |  |  |
| RRT | 1.08 (0.87, 1.34) | 0.469 |  |  |
| In-hospital medication |  |  |  |  |
| Inotrope use | 1.03 (0.85, 1.23) | 0.779 |  |  |
| Vasopressor use | 1.26 (1.04, 1.52) | 0.017 | 1.11 (0.92, 1.36) | 0.279 |

**Abbreviation**: CS: Cardiogenic shock; tCa: Total calcium; HR: Hazard ratio; CI: Confidence interval; ACS: Acute coronary symptom; CAD: Coronary artery disease; CHF: Chronic heart failure; PAD: Peripheral artery disease; DM: Diabetes mellitus; CKD: Chronic kidney disease; SBP: Systolic blood pressure; DBP: Diastolic blood pressure; MBP: Mean blood pressure; HR: Heart rate; tCa: Total calcium; AG: Anion gap; eGFR: estimated glomerular filtration rate; WBC: White blood cell; SOFA: Sequential Organ Failure Assessment; SAPS: Simplified Acute Physiology Score; GCS: Glasgow Coma Scale; RRT: Renal replacement treatment.

**Table S7.** The association between serum tCa levels and 30-day mortality in the subgroup analysis

|  |  | **Q1 (tCa＜7.8)** | **Q2 (7.8≤tCa＜8.3)** | **Q3 (8.3≤tCa＜8.9)** | **Q4 (8.9≤tCa)** |
| --- | --- | --- | --- | --- | --- |
| **Characteristics** | **N** | **HR (95% CI), *P* value** | **HR (95% CI), *P* value** | **Ref** | **HR (95% CI), *P* value** |
| Age |  |  |  |  |  |
| ≤72 | 446 | 1.40 (0.84, 2.34) 0.200 | 1.14 (0.69, 1.88) 0.616 | 1 | 0.97 (0.59, 1.60) 0.911 |
| ＞72 | 475 | 1.22 (0.86, 1.73) 0.267 | 0.66 (0.45, 0.95) 0.027 | 1 | 0.77 (0.53, 1.10) 0.145 |
| Sex |  |  |  |  |  |
| male | 555 | 1.31 (0.89, 1.92) 0.167 | 0.79 (0.54, 1.18) 0.250 | 1 | 1.13 (0.78, 1.62) 0.516 |
| female | 366 | 1.33 (0.85, 2.07) 0.214 | 0.89 (0.56, 1.41) 0.608 | 1 | 0.53 (0.32, 0.86) 0.010 |
| Current smoking |  |  |  |  |  |
| no | 449 | 1.51 (0.96, 2.37) 0.076 | 1.12 (0.72, 1.74) 0.613 | 1 | 0.97 (0.63, 1.51) 0.901 |
| yes | 472 | 1.24 (0.85, 1.80) 0.272 | 0.64 (0.42, 0.98) 0.041 | 1 | 0.77 (0.52, 1.14) 0.186 |
| Etiology |  |  |  |  |  |
| ACS | 620 | 1.53 (1.06, 2.20) 0.021 | 0.98 (0.68, 1.41) 0.897 | 1 | 0.87 (0.60, 1.27) 0.479 |
| Others | 301 | 1.03 (0.64, 1.67) 0.905 | 0.62 (0.36, 1.05) 0.073 | 1 | 0.76 (0.48, 1.21) 0.255 |
| CAD |  |  |  |  |  |
| no | 629 | 1.87 (1.09, 3.19) 0.023 | 0.95 (0.54, 1.67) 0.850 | 1 | 0.98 (0.58, 1.67) 0.948 |
| yes | 292 | 1.16 (0.82, 1.64) 0.396 | 0.80 (0.56, 1.13) 0.207 | 1 | 0.79 (0.56, 1.12) 0.192 |
| CHF |  |  |  |  |  |
| no | 716 | 1.23 (0.89, 1.71) 0.212 | 0.79 (0.56, 1.11) 0.170 | 1 | 0.83 (0.59, 1.16) 0.264 |
| yes | 205 | 1.74 (0.92, 3.31) 0.089 | 0.97 (0.51, 1.85) 0.938 | 1 | 0.86 (0.48, 1.54) 0.609 |
| AF |  |  |  |  |  |
| no | 476 | 1.19 (0.81, 1.75) 0.376 | 0.65 (0.42, 1.00) 0.052 | 1 | 0.66 (0.44, 0.99) 0.047 |
| yes | 445 | 1.52 (0.98, 2.36) 0.060 | 1.08 (0.71, 1.64) 0.730 | 1 | 1.09 (0.72, 1.65) 0.681 |
| Hypertension |  |  |  |  |  |
| no | 606 | 1.67 (1.18, 2.37) 0.004 | 0.85 (0.59, 1.23) 0.396 | 1 | 0.83 (0.58, 1.18) 0.295 |
| yes | 315 | 0.87 (0.51, 1.47) 0.592 | 0.81 (0.48, 1.36) 0.416 | 1 | 0.88 (0.53, 1.45) 0.612 |
| PAD |  |  |  |  |  |
| no | 775 | 1.32 (0.95, 1.82) 0.095 | 0.88 (0.63, 1.21) 0.425 | 1 | 0.66 (0.44, 0.99) 0.047 |
| yes | 146 | 1.48 (0.77, 2.81) 0.237 | 0.65 (0.29, 1.45) 0.293 | 1 | 1.09 (0.72, 1.65) 0.681 |
| DM |  |  |  |  |  |
| no | 588 | 1.67 (1.17, 2.41) 0.005 | 0.95 (0.65, 1.38) 0.775 | 1 | 0.83 (0.57, 1.22) 0.353 |
| yes | 333 | 0.89 (0.54, 1.46) 0.639 | 0.69 (0.42, 1.14) 0.147 | 1 | 0.86 (0.55, 1.34) 0.512 |
| CKD |  |  |  |  |  |
| no | 691 | 1.36 (0.98, 1.88) 0.063 | 0.85 (0.60, 1.20) 0.351 | 1 | 0.84 (0.60, 1.19) 0.330 |
| yes | 230 | 1.23 (0.63, 2.39) 0.538 | 0.81 (0.44, 1.47) 0.482 | 1 | 0.84 (0.49, 1.45) 0.533 |
| eGFR |  |  |  |  |  |
| ＜60 | 565 | 1.27 (0.91, 1.77) 0.158 | 0.72 (0.51, 1.03) 0.069 | 1 | 0.81 (0.58, 1.12) 0.202 |
| ＞60 | 356 | 1.67 (0.92, 3.01) 0.091 | 1.23 (0.68, 2.23) 0.498 | 1 | 0.90 (0.48, 1.68) 0.739 |

**Abbreviation**: CS: Cardiogenic shock; tCa: Total calcium; HR: Hazard ratio; CI: Confidence interval; ACS: Acute coronary symptom; CAD: Coronary artery disease; CHF: Chronic heart failure; AF: Atrial fibrillation; PAD: Peripheral artery disease; DM: Diabetes mellitus; CKD: Chronic kidney disease; eGFR: estimated glomerular filtration rate.

**Table S8.** Univariable and multivariable Cox regression analysis for serum iCa levels and 30-day mortality using original data

|  | **Univariable analysis** | | **Multivariable analysis** | |
| --- | --- | --- | --- | --- |
| **Characteristics** | **HR (95% CI)** | ***P* value** | **HR (95% CI)** | ***P* value** |
| Age | 1.04 (1.03, 1.06) | <0.001 | 1.04 (1.02, 1.06) | <0.001 |
| Sex |  |  |  |  |
| Male | 1 |  |  |  |
| Female | 1.06 (0.85, 1.31) | 0.529 |  |  |
| Ethnicity |  |  |  |  |
| Others | 1 |  |  |  |
| White | 0.83 (0.67, 1.03) | 0.085 |  |  |
| Current smoking | 1.07 (0.87, 1.34) | 0.631 |  |  |
| ACS etiology | 0.88 (0.70, 1.11) | 0.268 |  |  |
| Comorbidities |  |  |  |  |
| CAD | 1.06 (0.85, 1.34) | 0.704 |  |  |
| CHF | 0.90 (0.69, 1.18) | 0.367 |  |  |
| AF | 1.08 (0.85, 1.35) | 0.973 |  |  |
| Hypertension | 0.87 (0.68, 1.10) | 0.193 |  |  |
| PAD | 1.13 (0.80, 1.47) | 0.388 |  |  |
| Stroke | 0.63 (0.35, 1.11) | 0.106 |  |  |
| DM | 1.02 (0.84, 1.28) | 0.922 |  |  |
| CKD | 0.91 (0.69, 1.15) | 0.588 |  |  |
| Vital signs |  |  |  |  |
| SBP | 0.98 (0.97, 0.99) | <0.001 | 1.00 (0.99, 1.00) | 0.320 |
| DBP | 0.98 (0.97, 0.99) | 0.009 | 1.02 (1.01, 1.03) | 0.006 |
| MBP | 0.99 (0.98, 0.99) | <0.001 | 0.99 (0.98, 1.00) | 0.224 |
| HR | 1.00 (1.00, 1.00) | 0.341 |  |  |
| Laboratory-based data |  |  |  |  |
| iCa |  |  |  |  |
| Q1 (iCa＜1.04) | 1.72 (1.28, 2.31) | <0.001 | 1.36 (1.01, 1.85) | 0.047 |
| Q2 (1.04≤iCa＜1.11) | 1.05 (0.72, 1.48) | 0.557 | 0.93 (0.65, 1.29) | 0.700 |
| Q3 (1.11≤iCa＜1.17) | 1 |  | 1 |  |
| Q4 (1.17≤iCa) | 1.30 (0.98, 1.76) | 0.099 | 1.19 (0.87, 1.63) | 0.335 |
| Phosphorus | 1.20 (1.14, 1.28) | <0.001 | 1.16 (1.08, 1.22) | <0.001 |
| Potassium | 1.29 (1.07, 1.52) | 0.004 | 1.15 (0.94, 1.39) | 0.222 |
| Sodium | 0.99 (0.97, 1.01) | 0.499 |  |  |
| Chloride | 0.96 (0.94, 1.01) | 0.182 | 1.02 (0.97, 1.06) | 0.898 |
| Bicarbonate | 0.94 (0.92, 0.96) | <0.001 | 1.00 (0.98, 1.03) | 0.952 |
| Lactate | 1.20 (1.14, 1.25) | <0.001 | 1.18 (1.01, 1.26) | <0.001 |
| AG | 1.12 (9.98, 1.14) | 0.112 |  |  |
| PH | 0.63 (0.27, 1.41) | 0.214 |  |  |
| Creatinine | 1.10 (1.04, 1.15) | <0.001 | 0.99 (0.90, 1.12) | 0.771 |
| eGFR | 0.99 (0.98, 0.99) | <0.001 | 1.00 (0.99, 1.00) | 0.410 |
| Hemoglobin | 1.12 (0.98, 1.22) | 0.689 |  |  |
| Platelet | 1.00 (1.00, 1.00) | 0.487 |  |  |
| WBC | 1.03 (1.01, 1.06) | 0.141 |  |  |
| Scoring system |  |  |  |  |
| SOFA | 1.13 (1.10, 1.20) | 0.001 | 1.00 (0.96, 1.05) | 0.901 |
| SAPS II | 1.04 (1.03, 1.04) | <0.001 | 1.02 (1.01, 1.03) | 0.001 |
| GCS | 0.98 (0.95, 1.02) | 0.309 |  |  |
| Treatment information |  |  |  |  |
| Mechanical ventilation | 1.16 (0.91, 1.57) | 0.444 |  |  |
| RRT | 1.18 (0.88, 1.64) | 0.711 |  |  |
| In-hospital medication |  |  |  |  |
| Inotrope use | 1.11 (0.92, 1.63) | 0.322 |  |  |
| Vasopressor use | 1.30 (0.92, 1.63) | 0.422 |  |  |

**Abbreviation**: CS: Cardiogenic shock; iCa: Ionized calcium; HR: Hazard ratio; CI: Confidence interval; ACS: Acute coronary symptom; CAD: Coronary artery disease; CHF: Chronic heart failure; PAD: Peripheral artery disease; DM: Diabetes mellitus; CKD: Chronic kidney disease; SBP: Systolic blood pressure; DBP: Diastolic blood pressure; MBP: Mean blood pressure; HR: Heart rate; tCa: Total calcium; AG: Anion gap; eGFR: estimated glomerular filtration rate; WBC: White blood cell; SOFA: Sequential Organ Failure Assessment; SAPS: Simplified Acute Physiology Score; GCS: Glasgow Coma Scale; RRT: Renal replacement treatment.

**Table S9.** Univariable and multivariable Cox regression analysis for serum tCa levels and 30-day mortality using original data

|  | **Univariable analysis** | | **Multivariable analysis** | |
| --- | --- | --- | --- | --- |
| **Characteristics** | **HR (95% CI)** | ***P* value** | **HR (95% CI)** | ***P* value** |
| Age | 1.03 (1.02, 1.04) | <0.001 | 1.03 (1.02, 1.04) | <0.001 |
| Sex |  |  |  |  |
| Male | 1 |  |  |  |
| Female | 1.08 (0.88, 1.34) | 0.542 |  |  |
| Ethnicity |  |  |  |  |
| Others | 1 |  |  |  |
| White | 0.85 (0.67, 1.07) | 0.095 |  |  |
| Current smoking | 1.06 (0.86, 1.31) | 0.584 |  |  |
| ACS etiology | 0.88 (0.70, 1.11) | 0.211 |  |  |
| Comorbidities |  |  |  |  |
| CAD | 1.02 (0.79, 1.30) | 0.610 |  |  |
| CHF | 0.81 (0.59, 1.02) | 0.497 |  |  |
| AF | 1.02 (0.83, 1.25) | 0.873 |  |  |
| Hypertension | 0.78 (0.55, 1.01) | 0.202 |  |  |
| PAD | 1.16 (0.87, 1.52) | 0.401 |  |  |
| Stroke | 0.68 (0.37, 1.15) | 0.105 |  |  |
| DM | 1.01 (0.82, 1.26) | 0.919 |  |  |
| CKD | 0.90 (0.65, 1.15) | 0.512 |  |  |
| Vital signs |  |  |  |  |
| SBP | 0.98 (0.97, 0.99) | <0.001 | 1.00 (0.99, 1.00) | 0.315 |
| DBP | 0.99 (0.98, 0.99) | 0.013 | 1.02 (1.01, 1.03) | 0.065 |
| MBP | 0.99 (0.98, 0.99) | <0.001 | 0.99 (0.98, 1.00) | 0.185 |
| HR | 1.00 (0.99, 1.00) | 0.303 |  |  |
| Laboratory-based data |  |  |  |  |
| tCa, |  |  |  |  |
| Q1 (tCa＜7.8) | 1.35 (1.01, 1.81) | 0.049 | 1.32 (0.97, 1.84) | 0.107 |
| Q2 (7.8≤tCa＜8.3) | 0.82 (0.60, 1.10) | 0.247 | 0.75 (0.54, 1.02) | 0.087 |
| Q3 (8.3≤tCa＜8.9) | 1 |  | 1 |  |
| Q4 (8.9≤tCa) | 0.87 (0.65, 1.21) | 0.344 | 0.79 (0.59, 1.11) | 0.092 |
| Phosphorus | 1.21 (1.14, 1.30) | <0.001 | 1.16 (1.09, 1.25) | <0.001 |
| Potassium | 1.29 (1.05, 1.52) | 0.002 | 1.08 (0.88, 1.31) | 0.500 |
| Sodium | 0.99 (0.97, 1.01) | 0.479 |  |  |
| Chloride | 0.99 (0.97, 1.00) | 0.049 | 1.00 (0.98, 1.01) | 0.581 |
| Bicarbonate | 0.99 (0.94, 1.04) | 0.503 |  |  |
| Lactate | 1.23 (1.17, 1.33) | <0.001 | 1.12 (1.06, 1.18) | <0.001 |
| AG | 1.08 (0.97, 1.09) | 0.073 |  |  |
| PH | 0.62 (0.28, 1.37) | 0.234 |  |  |
| Creatinine | 1.10 (1.04, 1.15) | <0.001 | 1.00 (0.90, 1.11) | 0.989 |
| eGFR | 0.99 (0.99, 0.99) | 0.001 | 1.00 (0.99, 1.00) | 0.354 |
| Hemoglobin | 1.04 (0.98, 1.12) | 0.502 |  |  |
| Platelet | 1.00 (1.00, 1.00) | 0.387 |  |  |
| WBC | 1.03 (0.99, 1.12) | 0.245 |  |  |
| Scoring system |  |  |  |  |
| SOFA | 1.11 (1.07, 1.16) | <0.001 | 1.00 (0.96, 1.05) | 0.781 |
| SAPS II | 1.03 (1.03, 1.04) | 0.002 | 1.02 (1.01, 1.03) | 0.001 |
| GCS | 0.98 (0.95, 1.03) | 0.219 |  |  |
| Treatment information |  |  |  |  |
| Mechanical ventilation | 1.12 (0.87, 1.46) | 0.299 |  |  |
| RRT | 1.11 (0.86, 1.44) | 0.370 |  |  |
| In-hospital medication |  |  |  |  |
| Inotrope use | 1.16 (0.90, 1.53) | 0.431 |  |  |
| Vasopressor use | 1.35 (0.99, 1.53) | 0.106 |  |  |

**Abbreviation**: CS: Cardiogenic shock; tCa: Total calcium; HR: Hazard ratio; CI: Confidence interval; ACS: Acute coronary symptom; CAD: Coronary artery disease; CHF: Chronic heart failure; PAD: Peripheral artery disease; DM: Diabetes mellitus; CKD: Chronic kidney disease; SBP: Systolic blood pressure; DBP: Diastolic blood pressure; MBP: Mean blood pressure; HR: Heart rate; tCa: Total calcium; AG: Anion gap; eGFR: estimated glomerular filtration rate; WBC: White blood cell; SOFA: Sequential Organ Failure Assessment; SAPS: Simplified Acute Physiology Score; GCS: Glasgow Coma Scale; RRT: Renal replacement treatment.
